# Outcome of aggressive B-cell lymphoma with *TP53* alterations administered with CAR T-cell cocktail alone or in combination with ASCT

**DOI:** 10.1038/s41392-022-00924-0

**Published:** 2022-04-11

**Authors:** Jia Wei, Min Xiao, Zekai Mao, Na Wang, Yang Cao, Yi Xiao, Fankai Meng, Weimin Sun, Ying Wang, Xingcheng Yang, Liting Chen, Yicheng Zhang, Haichuan Zhu, Shangkun Zhang, Tongcun Zhang, Jianfeng Zhou, Liang Huang

**Affiliations:** 1 Department of Hematology, Tongji Hospital, Tongji Medical College, Huazhong University of Science and Technology, Wuhan, Hubei 430030 China; 2 Immunotherapy Research Center for Hematologic Diseases of Hubei Province, Wuhan, Hubei 430030 China; 3 College of Life Science and Health, Wuhan University of Science and Technology, Wuhan, Hubei 430065 China; 4 Wuhan Bio-Raid Biotechnology CO., LTD, Wuhan, Hubei 430078 China

**Keywords:** Haematological cancer, Haematological cancer

## Abstract

*TP53* gene alteration confers inferior prognosis in refractory/relapse aggressive B-cell non-Hodgkin lymphoma (r/r B-NHL). From September 2016 to September 2020, 257 r/r B-NHL patients were assessed for eligibility for two trials in our center, assessing anti-CD19 and anti-CD22 chimeric antigen receptor (CAR19/22) T-cell cocktail treatment alone or in combination with autologous stem cell transplantation (ASCT). *TP53* alterations were screened in 123 enrolled patients and confirmed in 60. CAR19/22 T-cell administration resulted in best objective (ORR) and complete (CRR) response rate of 87.1% and 45.2% in patients with *TP53* alterations, respectively. Following a median follow-up of 16.7 months, median progression-free survival (PFS) was 14.8 months, and 24-month overall survival (OS) was estimated at 56.3%. Comparable ORR, PFS, and OS were determined in individuals with or without *TP53* alterations, and in individuals at different risk levels based on functional stratification of *TP53* alterations. CAR19/22 T-cell treatment in combination with ASCT resulted in higher ORR, CRR, PFS, and OS, but reduced occurrence of severe CRS in this patient population, even in individuals showing stable or progressive disease before transplantation. The best ORR and CRR in patients with *TP53* alterations were 92.9% and 82.1%, respectively. Following a median follow-up of 21.2 months, 24-month PFS and OS rates in patients with *TP53* alterations were estimated at 77.5% and 89.3%, respectively. In multivariable analysis, this combination strategy predicted improved OS. In conclusion, CAR19/22 T-cell therapy is efficacious in r/r aggressive B-NHL with *TP53* alterations. Combining CAR-T cell administration with ASCT further improves long-term outcome of these patients.

## Introduction

Individuals with aggressive B-cell Non-Hodgkin’s Lymphoma (B-NHL) in either primary refractory or relapse status have poor prognosis. Salvage high-dose chemotherapy (HDT) with subsequent autologous stem cell transplantation (ASCT) is considered the standard therapeutic option for chemosensitive cases in the rituximab era^1,2^. However, in cases resistant to chemotherapy, the role of ASCT remains controversial and this salvage regimen does not show additional benefits, indicating a need for treatments with higher efficacy. Recently, chimeric antigen receptor (CAR) T-cell treatment has achieved unprecedented response in r/r B-NHL. In ZUMA-1, axicabtagene ciloleucel (Axi-cel), a first-in-class CD19-targeted CAR (CAR19) T-cell product, achieved an objective response rate (ORR) of 82% and a median overall survival (OS) greater than 2 years in r/r aggressive B-NHL following ≥2 lines of systemic therapies. Following a median follow-up of 55.1 months, median OS time and 4-year OS rate were 25.8 months, and 44%, respectively^3,4^. In particular, in high-risk cases, CAR T-cell treatment offers potential solutions^5^. In ZUMA-12, Axi-cel was administered as first-line treatment in high-risk large B-cell lymphoma (LBCL) patients, of whom about 60% had double- or triple-hit lymphoma (DHL/THL). An interim analysis indicated Axi-cel has significant clinical benefits in these unfavorable patients, with high treatment response rate and a manageable safety profile^6^.

However, in *TP53*-disrupted B-cell malignancies, CAR T-cell treatment has yielded controversial results. We previously reported an acute B-cell lymphoblastic lymphoma case who achieved minimal residual disease (MRD)-negative CR for 15 months after receiving a CAR19/22 T-cell treatment in the context of Li-Fraumeni syndrome, a rarely diagnosed autosomal dominant condition predisposing the carrier to malignant disease and commonly associated with germline *TP53* mutations^7^. This study suggested a potentially effective role for CAR T-cell treatment in such patients^7^. However, relapse remains a challenge in patients with *TP53*-disrupted r/r B-cell acute lymphoblastic leukemia (B-ALL) after receiving CAR19 T-cell therapy, even consolidated with allogeneic hematopoietic stem cell transplantation^8,9^. The effect of CAR-T cell treatment, especially CAR-T with dual-targeting strategies, in r/r B-NHL cases with *TP53* alterations remains unclear and needs to be systematically investigated.

The evolutionarily conserved *TP53* gene, also called the ‘guardian of the genome’, represents a key tumor suppressor gene regulating cell cycle arrest, DNA repair pathways, apoptotic death, senescence, and autophagy in cells under stress conditions^10–12^. In humans, *TP53* is the most frequently disrupted gene in malignant diseases being altered in 20–25% of individuals with aggressive B-NHL^13–15^. A higher incidence of *TP53* mutation is likely to be found in r/r B-NHL^16,17^. Meanwhile, genomic instability caused by *TP53* alterations induces chemoresistance and progression in cancer^18,19^. Multiple reports have shown disruption of *TP53* is involved in lymphomagenesis and treatment resistance, and increased upon relapse in diffuse large B-cell lymphoma (DLBCL) and other lymphoid malignancies^20–22^. In DLBCL, *TP53* gene alterations, including *TP53* mutations and/or del(17p), are detected in approximately 30–50% of patients and mostly result in inferior outcome upon treatment with rituximab plus cyclophosphamide, hydroxydaunorubicin, vincristine, and prednisone (R-CHOP)^13,14,23^. However, even after high-dose therapy (HDT)/ASCT, the prognosis of these patients remains unfavorable. In the multicenter, open label, randomized, phase 3 DLCL04 trial, individuals with untreated DLBCL at high-risk were consolidated with HDT/ASCT as first-line treatment^24^. As the result, 5-year OS rates were 81 and 33% in wild type (*TP53*wt) and mutant (*TP53*mut) *TP53* cases, respectively. The 5-year failure-free survival (FFS) rates were 73% for *TP53*wt patients and 19% for *TP53*mut cases^25^. Similarly, patients with *TP53*-disrupted mantle cell lymphoma (MCL) or Burkitt lymphoma (BL) may not benefit from intensive chemotherapy, ASCT, or other targeted treatment strategies^26–29^. Due to the poor outcome conferred by *TP53* alterations, attempts to manage these patients are unfruitful^30^.

The safety and efficacy of two clinical studies examining CAR19/22 T-cell treatment alone (Trial A) or combined with ASCT (Trial B) in B-cell malignancies were examined in our recent report^31,32^. To assess the prognostic effect of *TP53* gene disruption and develop a therapeutic strategy for these patients, the present work systemically assessed the efficacy and safety of these 2 regimens in this patient population with increased sample size and follow-up time.

## Results

### Enrollment and baseline features

From September 2016 to September 2020, 257 r/r B-NHL patients were assessed for eligibility for two trials. Supplemental Fig. 1 shows the schematic diagrams of the study procedures in both trials. *TP53* alterations, including *TP53* mutation and del(17p), were screened in 123 enrolled patients and confirmed in 60. Supplemental Fig. 2 shows a flowchart of patient enrollment and group assignment. In Trial A, 32 patients (48.5%) carried *TP53* alterations (group B), while 34 (51.5%) did not (group A). In Trial B, 28 patients (49.1%) carried *TP53* alterations (group C), while 29 (50.9%) did not (group D). The cutoff date for follow-up was April 30, 2021.

The patient baseline features are listed in Table 1. All features were balanced among the 4 groups (Table 1). Of the patients with *TP53* alterations in both trials, 7 had prior ASCT, including 1 in Trial B. All had measurable disease, and 11 (18.3%) showed a stable (SD) or progressive (PD) disease at enrollment. Of note, although showing a SD/PD at enrollment, 5 patients (17.9% in group C) were enrolled in Trial B, a study involving ASCT.

**Table 1:** Patients’ characteristics

Characteristics	Values				*P*
	Group A, Trial A (W/O *TP53* Alterations)	Group B, Trial A (With *TP53* Alterations)	Group C, Trial B (With *TP53* Alterations)	Group D, Trial B (W/O TP53 Alterations)	
Patients number	34	32	28	29	
Age, median (range)	50 (17–69)	47 (29–63)	40 (23–61)	39 (24–61)	0.070
Gender (M/F)	21/13	22/10	18/10	16/13	0.742
ECOG PS					0.386
0–1	20 (58.8%)	22 (68.8%)	16 (57.1%)	22 (75.9%)	
2	14 (41.2%)	10 (31.3%)	12 (42.8%)	7 (24.1%)	
Pathologic subtype					0.237
DLBCL, NOS	20 (58.8%)	26 (81.2%)	18 (64.3%)	22 (75.9%)	
DLBCL-tFL	6 (17.6%)	3 (9.4%)	4 (14.3%)	4 (13.8%)	
HGBL DH/TH	5 (14.7%)	0 (0.0%)	2 (7.1%)	3 (10.3%)	
HGBL, NOS	2 (5.8%)	1 (3.1%)	0 (0.0%)	0 (0.0%)	
Burkitt lymphoma	1 (2.9%)	1 (3.1%)	2 (7.1%)	0 (0.0%)	
MCL	0 (0.0%)	1 (3.1%)	0 (0.0%)	0 (0.0%)	
Others^**a**^	0 (0.0%)	0 (0.0%)	2 (7.1%)	0 (0.0%)	
Tumor mass					0.160
≥5 cm	13 (38.2%)	16 (50.0%)	7(25.0%)	8 (27.6%)	
Disease status					0.409
Primary refractory	6 (17.6%)	12 (37.5%)	8 (28.6%)	12 (41.4%)	
First relapse	16 (47.0%)	6 (18.8%)	10 (35.7%)	10 (14.5%)	
Second relapse	8 (23.5%)	9 (28.1%)	7 (25.0%)	4 (13.8%)	
≥ 3rd relapse	4 (11.8%)	5 (15.6%)	3 (10.7%)	3 (10.3%)	
No. of treatment lines					0.174
2	10 (29.4%)	3 (9.4%)	8 (28.6%)	4 (13.8%)	
3	13 (38.2%)	10 (31.3%)	11 (39.3%)	11 (37.9%)	
≥4	11 (32.4%)	19 (59.4%)	9 (32.1%)	14 (48.3%)	
Previous ASCT	6 (17.6%)	6 (18.8%)	1 (3.6%)	1 (3.4%)	0.088
Previous CART	0 (0%)	0 (0%)	3 (10.7%)	1 (3.4%)	0.066
Bridging treatment^b^	8 (23.5%)	9 (28.1%)	3 (10.7%)	3 (10.3%)	0.180
Baseline LDH ratio					0.380
<1×ULN	14 (41.2%)	11 (34.4%)	9 (32.1%)	10 (14.5%)	
1–3×ULN	16 (47.0%)	18 (56.2%)	11 (39.3%)	16 (55.2%)	
≥4×ULN	4 (11.8%)	3 (9.4%)	8 (28.6%)	3 (10.3%)	
Disease stage^c^					0.647
I or II	6 (17.6%)	4 (12.5%)	4 (14.3%)	7 (24.1%)	
III or IV	28 (82.4%)	28 (87.5%)	24 (85.7%)	22 (75.9%)	
Response at enrollment					0.796
PR	27 (79.4%)	26 (81.2%)	23 (82.1%)	21 (72.4%)	
PD/SD	7 (20.6%)	6 (18.8%)	5 (17.9%)	8 (27.6%)	
IPI score					0.800
0–2	15 (44.1%)	13 (40.6%)	10 (35.7%)	14 (48.3%)	
3–4	19 (55.9%)	19 (59.4%)	18 (64.3%)	15 (51.7%)	
Cell of origin (*n* = 113)					0.520
GCB	13 (38.2%)	14 (43.8%)	8 (28.6%)	9 (31.0%)	
non-GCB	18 (52.9%)	15 (46.9%)	16 (57.1%)	20 (69.0%)	
Dosage of CAR T cells (×10^6^/kg)					
CAR19, median (range)	4.0 (1.4–7.7)	4.9 (1.9–8.9)	4.0 (2.1–12.6)	4.0 (1.0–10.0)	0.164
CAR22, median (range)	5.9 (2.1–10.0)	6.0 (1.0–11.4)	4.0 (2.1–6.3)	4.0 (0.8–10.0)	0.074

^a^Others, including 1 with B-cell lymphoma, unclassified with properties between DLBCL and classical Hodgkin lymphoma, and 1 with B-cell lymphoblastic lymphoma;

^b^Disease stage based on the modified Ann Arbor staging system. *W/O* without, *ECOG PS* Eastern Cooperative Oncology Group (ECOG) performance status, *DLBCL NOS* diffuse large B-cell lymphoma, not otherwise specified, *DLBCL-tFL* diffuse large B-cell lymphoma transformed from follicular lymphoma (FL), *HGBL DH/TH* high-grade B-cell lymphoma with *MYC* and *BCL2* and/or *BCL6* rearrangements, *HGBL NOS* HGBL, not otherwise specified, *MCL* mantle cell lymphoma, *ASCT* autologous stem cell transplantation;

^**c**^Bridging treatment, including radiotherapy, ibrutinib, glucocorticoid, sequential ifosfamide and etoposide, and rituximab et al between hematopoietic stem cell & T cell collection and lymphodepletion or myeloablative chemotherapy before infusion of CART, *LDH* lactate dehydrogenase, *ULN* upper limit of normal, *PR* partial response, *SD* stable disease, *PD* progressive disease, *IPI* international prognostic index, *GCB* germinal center B-cell like.

### Profiles of TP53 alterations

**Fig. 1:**
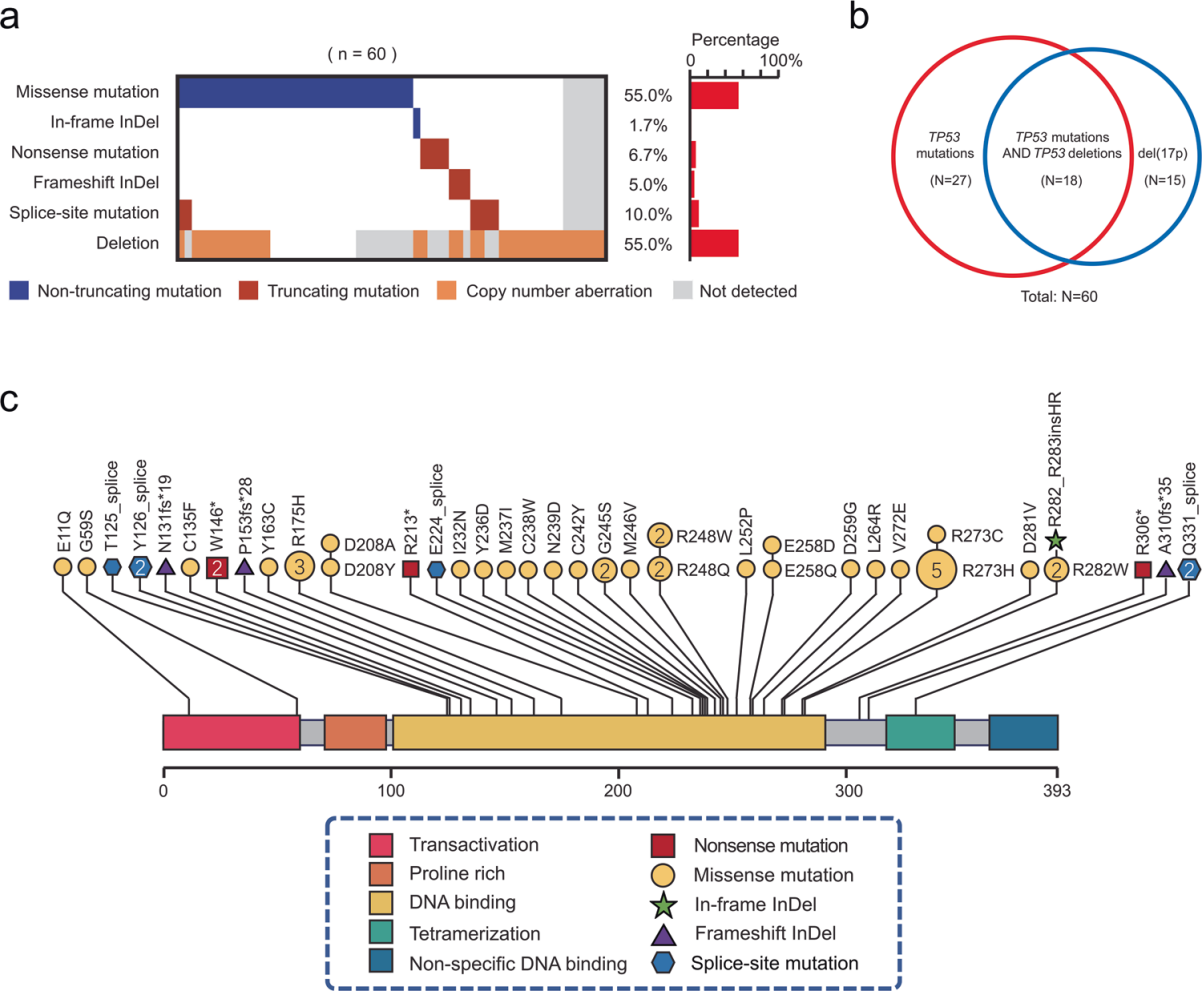
Mutational profile of TP53 alterations. **a** Heatmap showing individual non-synonymous somatic mutations found in individuals administered CAR-T cell infusion (*n* = 60). Each row represents one type of *TP53* alterations, and each column represents a patient with R/R B-NHL. The horizonal bar graph shows gene mutation frequencies. **b** Venn diagram demonstrates different *TP53* alterations identified in 60 cases. Totally 42 (70.0%) individuals harbored *TP53* mutation(s) or sole del(17p), with 27 (45.0%) and 15 (25.0%) showing *TP53* mutation(s) and deletions, respectively. The remaining 18 (30.0%) cases had biallelic *TP53* aberrations (*TP53* mutation and del(17p) in the first and second alleles, respectively). **c** *TP53* mutations are plotted above the protein divided into the main domains. Among the 51 mutations identified, 37 were missense mutations (yellow), 6 were splice-site mutations (blue), 4 were nonsense mutations (red), 3 were frameshift insertions or deletions (indels; purple), and 1 was a non-frameshift indel (green). Recurrent mutations were identified in 3 codons, including R273 (*n* = 6), R248 (*n* = 4) and R175 (*n* = 3). The *x*-axis indicates the amino acid position

Heterozygous gene mutations and/or deletions in *TP53* are depicted in Fig. 1a. Totally 42 (70.0%) individuals harbored *TP53* mutation(s) or sole del(17p), with 27 (45.0%) and 15 (25.0%) showing *TP53* mutation(s) and deletions, respectively (Fig. 1b). The remaining 18 (30.0%) cases had biallelic *TP53* aberrations. Among the 51 mutations identified, 45 (88.0%) were in the DNA-binding domain (DBD; amino acids 101–294), as reflected in Fig. 1c. Compound heterozygous mutations were found in 6 patients (10.0%). Germline *TP53* variations were validated in 2 patients (3.3%). In total, 40 (66.7%) cases had loss-of-function alterations inactivating *TP53*, with decreased copy number loss or truncations. Comparable *TP53* mutation patterns were observed based on treatment strategies (trial A *vs* trial B) or different clinical outcomes (responders vs non-responders or no relapse *vs* relapse) (Supplemental Fig. 3).

### Infusion and kinetics of CAR T cells and engraftment of hematopoietic stem cells (HSCs)

Patients in Trial A received 4.1 (1.4–8.9) × 10^6^/kg CAR19 T cells and 6.0 (1.0–11.4) × 10^6^/kg CAR22 T cells, and those in Trial B received similar doses, i.e., 4.0 (1.0–12.6) × 10^6^/kg CAR19 T cells and 4.0 (0.8–10.0) × 10^6^/kg CAR22 T cells. Median manufacturing times were 13 days (range: 8–20 days) and 12 days (range: 9–22 days) for CAR19 T and CAR22 T cells, respectively. Transduction efficiencies for CAR19 and CAR22 T cells averaged 46.2 ± 19.6% and 47.6 ± 20.4%, respectively. The mean viability rates of CAR-T cells were 92.7 ± 2.5% for CAR19 T cells and 93.1 ± 3.4% for CAR22 T cells. The CD4/CD8 ratios for CAR19 T and CAR22 T cells were 1.8 ± 0.1 and 1.8 ± 0.2 in totally 37 cases, respectively. Cell dosages (Table 1), median times to reach peak expansion, and median copies of peak expansion for CAR19 and CAR22 T cells in peripheral blood were similar between different groups in both 2 trials, suggesting comparable kinetics and expansion of CAR19 and CAR22 T cells in both trials (Supplemental Fig. 4). Additionally, the median dosage of autologous stem cells (CD34^+^ cells) in trial B was 7.4 (2.2–13.0) × 10^6^/kg. Administration of the CAR19/22 T-cell cocktail was generally initiated at day +3 (range: days +2 to +6). All trial B patients had successful HSC engraftment. The median durations of neutrophil or platelet engraftment were 12 days (range: 7–33 days) and 16 days (range: 9–42 days), respectively.

### Treatment effects of the CAR T-cell cocktail

**Fig. 2:**
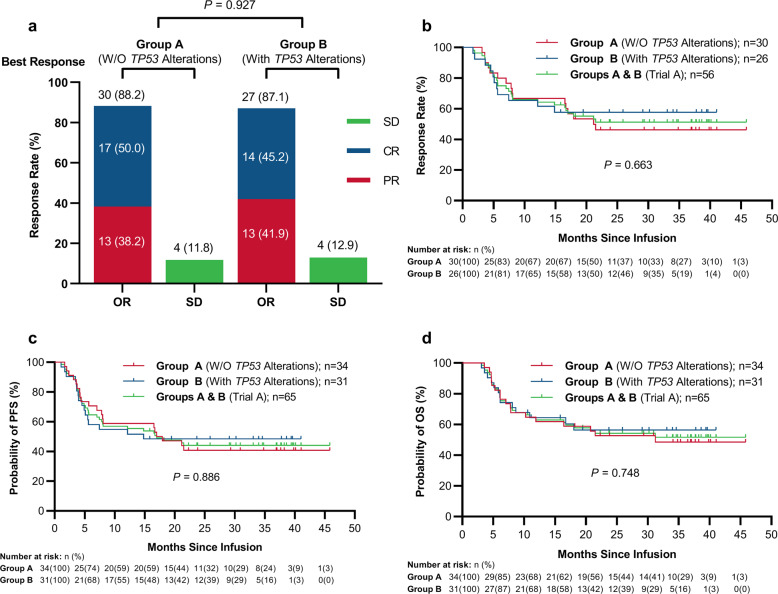
Outcomes of r/r aggressive B-cell lymphoma cases according to TP53 alteration status after CAR19/22 T-cell infusion. **a** Best responses in r/r aggressive B-cell lymphoma cases with or without *TP53* alterations. Kaplan–Meier analysis of DOR (**b**), PFS (**c**), and OS (**d**) in r/r aggressive B-cell lymphoma cases on the basis of *TP53* alteration status. ORR, objective response rate; OR objective response; CR complete response; PR partial response; SD stable disease; DOR duration of response; W/O without

In Trial A, 57 cases (87.7%, 95% CI: 77.6–93.6%) achieved best objective response (OR) to CAR19/22 T-cell therapy, including 31 (47.7%, 95% CI: 36.0–59.6%) with a CR (Supplemental Fig. 5). There was no marked difference in best OR rate (ORR, CR + PR) between cases with or without *TP53* disruption (87.1% in group B vs. 88.2% in group A, *P* = 0.927, Fig. 2a). At the last follow up, 15 cases (48.4%, 95% CI: 32.0–65.2%) maintained their initial responses while 16 (51.6%, 95% CI: 34.8–68.0%) experienced disease progression.

**Fig. 3:**
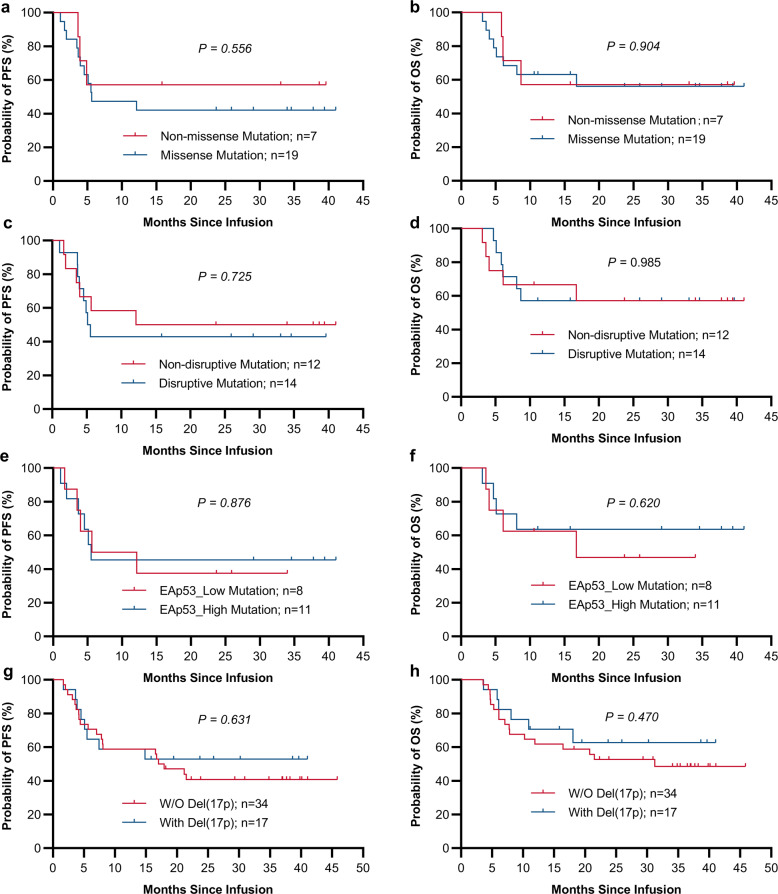
Outcomes of r/r aggressive B-cell cases stratified by four functional classification systems of TP53 alterations after CAR19/22 T-cell infusion. In individuals with r/r aggressive B-cell lymphoma and concurrent *TP53* mutations, PFS (**a**, **c** and **e**) and OS (**b**, **d**, and **f**) were compared according to functional classifications of *TP53* mutations. Kaplan–Meier analysis of PFS (**a**) and OS (**b**) for the non-missense and missense mutation groups. Kaplan–Meier analysis of PFS (**c**) and OS (**d**) for the non-disruptive and disruptive mutation groups. Kaplan–Meier analysis of PFS (**e**) and OS (**f**) for EAp53 low-risk (<75) and EAp53 high-risk (≥75) mutation groups. Kaplan–Meier analysis of PFS (**g**) and OS (**h**) in the del(17p) and non-del(17p) groups

In patients with disrupted *TP53* (group B), following a median follow-up of 16.7 months (range: 3.1–41.0 months), median DOR and OS were not reached (Fig. 2b, d). Median PFS was 14.8 months (95%CI: 5.1–NE) (Fig. 2c). In these cases, 24-month PFS and OS estimates were 48.4% (95% CI: 30.2–64.4%) and 56.3% (95% CI: 36.6–72.0%), respectively (Fig. 2c, d). Of note, DOR, PFS, and OS were comparable in the wild type and *TP53* alteration groups when treated with CAR19/22 T-cell cocktail therapy (Fig. 2b–d). In addition, similar trends of best ORR, DOR, and survival were exhibited among the 54 patients in Trial A who had r/r DLBCL, irrespective of *TP53* gene status (Supplemental Fig. 6a–d). Similarly, in each functional classification system of *TP53* alterations, best ORR (Supplemental Fig. 7a) and survival features (Fig. 3a–h) were comparable in cases with different risk levels, suggesting CAR19/22 T-cell treatment overcame the negative effects of *TP53* disruption in aggressive B-cell lymphoma.

### Superior effects of ASCT incorporating CAR19/22 T-cell treatment

**Fig. 4:**
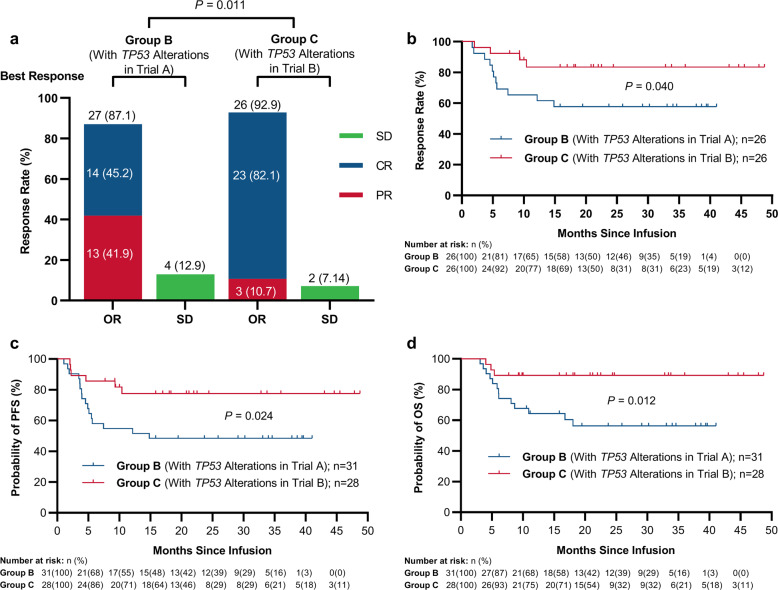
Outcomes of r/r aggressive B-cell lymphoma cases with concurrent TP53 alterations after CAR19/22 T-cell infusion alone or combined with ASCT. **a** Best responses after CAR19/22 T-cell infusion alone or combined with ASCT. Kaplan-Meier analysis of DOR (**b**), PFS (**c**), and OS (**d**) is shown

**Fig. 5:**
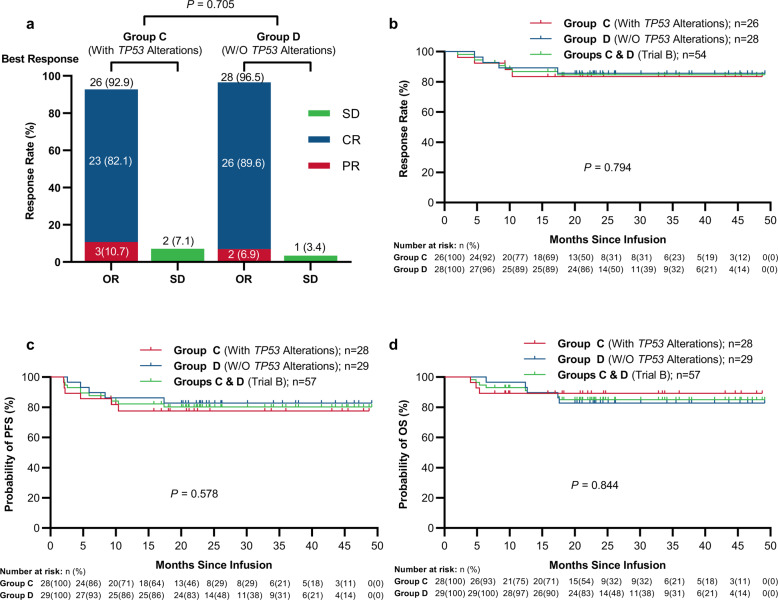
Outcomes of r/r aggressive B-cell lymphoma cases based on TP53 alteration status after ASCT combined with CAR19/22 T-cell infusion. **a** Best responses in r/r aggressive B-cell lymphoma cases with or without *TP53* alterations. Kaplan–Meier analysis of DOR (**b**), PFS (**c**), and OS (**d**) is shown. ORR objective response rate; OR objective response; CR complete response; PR partial response; SD stable disease; DOR duration of response; W/O without

Among the 28 individuals with r/r aggressive B-NHL and concurrent *TP53* alterations (group C in Trial B), 26 (92.9%, 95%CI: 77.4–98.7%) showed best ORR when treated with ASCT incorporating CAR19/22 T-cell cocktail treatment (Trial B), including 23 (82.1%, 95%CI: 64.4–92.1%) CR cases (Fig. 4a). After a median follow-up of 21.2 (range: 4.0–48.7) months, median DOR, PFS, and OS were not reached (Fig. 4b–d). In these cases, 24-month PFS and OS estimates were 77.5% (95% CI: 56.5–89.3%) and 89.3% (95% CI: 70.4–96.4%) (Fig. 4c, d), respectively. At the last follow-up, 22 cases (78.6%, 95%CI: 60.5–89.8%) maintained the initial responses while 6 (21.4%, 95%CI: 10.2–39.5%) had disease progression. Surprisingly, in comparison with CAR19/22 T-cell cocktail treatment (group B), marked increased DOR (*P* = 0.040, Fig. 4b), PFS (*P* = 0.024, Fig. 4c) and OS (*P* = 0.012, Fig. 4d) were observed after CAR T-cell therapy incorporation in ASCT (group C). Similarly, comparable best ORR, DOR, PFS, and OS were detected in the wild type and *TP53* alteration groups administered ASCT incorporated with CAR19/22 T-cell cocktail infusion (Fig. 5a–d). In each functional classification system of *TP53* alterations, best ORR (Supplemental Fig. 7b) had no marked difference in trial B cases of distinct risk levels.

Likewise, in patients with r/r DLBCL and concurrent *TP53* alterations, superior best response (*P* < 0.001, Supplemental Fig. 8a) and DOR (*P* = 0.039, Supplemental Fig. 8b), and trends for better PFS (*P* = 0.086, Supplemental Fig. 8c) and OS (*P* = 0.076, Supplemental Fig. 8d) were demonstrated when administered ASCT incorporating CAR19/22 T-cell cocktail infusion. Of note, in cases with a SD/PD at enrollment in Trial B, best ORR, and best CR rate were 100% (95%CI: 56.6–100%) and 80.0% (95%CI: 37.6–99.0%), respectively. Similar PFS (*P* = 0.892, Supplemental Fig. 9a) and OS (*P* = 0.510, Supplemental Fig. 9b) were obtained in patients with a PR or PD/SD at enrollment, suggesting that even in patients with a SD/PD at enrollment, durable remission could be obtained with CAR T-cell therapy incorporation in ASCT.

### Predictive factors for response and survival

In cases with disrupted *TP53*, those with maintained OR at month 3 (R3m, i.e., “durable complete or partial response at month 3) (responders) had significantly extended PFS (*P* < 0.001, Supplemental Fig. 10a) and OS (*P* < 0.001, Supplemental Fig. 10b) when compared with patients who failed to have R3m (non-responders). Similarly, improved PFS and OS were found in cases with R6m compared to those who failed to have R6m (Supplemental Fig. 10c, d). Compared with non-responders, responders tended to have more persistent CAR19 (75.0% vs. 33.3%, *P* = 0.047) and CAR22 (87.5% vs. 31.4%, *P* = 0.0004) transgenes (Supplemental Fig. 11a) at month 3. Accordingly, fewer non-responders showed durable B-cell aplasia (BCA) at month 3 than responders (62.5% vs. 94.1%, *P* = 0.028, Supplemental Fig. 11b). Different *TP53* alteration patterns exhibited comparable ORRs at month 3 (Supplemental Table 1). However, R3m was affected by CRS severity (≥ grade 3) after CAR19/22 T-cell treatment (*P* = 0.013, Supplemental Table 2), while being consistent in different subgroups administered ASCT incorporating CAR19/22 T-cell cocktail infusion (Supplemental Table 3).

Additionally, survival-associated clinical features were identified by univariable and multivariable analyses in individuals with aggressive r/r B-NHL and concurrent *TP53* alterations (Table 2). OS was remarkably associated with R3m (HR14.50, 95%CI: 4.3–49.1, *P* < 0.0001), enrolled trial (HR 4.40, 95% CI: 1.2–16.0, *P* = 0.025) and performance status (HR 1.17, 95% CI: 1.0–3.1, *P* = 0.0498) in multivariable analysis (Table 2). In log-rank survival analysis, OS was significantly improved in patients who showed R3m (*P* < 0.0001, Supplemental Fig. 10b), received ASCT incorporating CAR19/22 T-cell cocktail treatment (*P* = 0.012, Fig. 4d), or showed an Eastern Cooperative Oncology Group performance status (ECOG PS) of 0–1 (*P* = 0.011, Supplemental Fig. 12).

**Table 2:** Univariable and multivariable analysis of OS

Variables	Univariable			Multivariable		
	HR	95%CI	*P*	HR	95%CI	*P*
R3m: responder vs. non-responder	22.19	6.86–71.81	<0.0001	14.50	4.28–49.08	<0.0001
LDH: < 1×ULN vs.1–3×ULN vs. >3×ULN	2.02	0.98–4.15	0.056			
Tumor mass: <5 cm vs. ≥5 cm	1.91	0.72–5.11	0.194			
CRS: 0–2 vs. ≥3	1.85	1.13–3.04	0.015			
Treatment option: Trial A vs. Trial B	4.36	1.24–15.31	0.022	4.40	1.21–16.02	0.025
ECOG PS: 0–1 vs. 2	1.86	1.12–3.10	0.016	1.17	1.00–3.05	0.0498

*ULN* upper limit of normal, *HR* hazard ratio, *CI* confidence interval, *LDH* lactate dehydrogenase, *CRS* cytokine release syndrome, *ECOG PS* Eastern Cooperative Oncology Group Performance Status

### Safety profiles

Comparable safety profiles were observed in both trials (Supplemental Table 4). The commonest adverse events were cytopenia, including neutropenia, lymphopenia, thrombocytopenia, and anemia. Anemia and severe thrombocytopenia were more common in Trial B than in Trial A due to 2 different conditioning regimens. No marked differences in other adverse effects were found between these 2 trials. Infection of grade 3 or higher was found in 4.54% Trial A and 7.14% Trial B cases. One patient in Trial A had grade 5 infection (invasive fungal infection) and succumbed to septic shock within 30 days.

Most patients in both groups developed CRS, with 90.9% (95%CI: 81.6–95.8%) in Trial A and 94.7% (95%CI: 85.6–98.6%) in Trial B (Supplemental Table 4). In Trial A cases, median onset of CRS occurred on day 4 (range: 2–8 days), with median resolution on day 10 (range: 6–24 days) post-infusion. In Trial B, median onset of CRS occurred on day 5 (range: 3–10 days), with median resolution on day 9 (range: 5–20 days). Group C patients had decreased incidence of severe CRS (grade ≥3) compared with group B cases (10.7% vs. 37.5%, *P* = 0.020, Supplemental Fig. 13). Similar incidence rates of immune effector cell-associated neurotoxicity syndrome (ICANS) were obtained in both trials, with 9.1% (95% CI: 4.2–18.4%) in Trial A and 19.3% (95% CI: 11.1–31.3%) in Trial B. In both trials, no patients died of CRS or developed severe ICANS (grade ≥3).

## Discussion

*TP53* represents the top candidate gene frequently disrupted in aggressive B-cell lymphomas including r/r DLBCL. Its alterations are implicated in treatment failure and remain clonally stable following multiple lines of therapies, suggesting a role in primary chemoresistance, and therefore have close correlations with disease progression and dismal survival rates. Salvage platinum-based chemotherapy with subsequent HDT/ASCT is presently considered as the standard treatment option for second-line chemo-sensitive patients. However, even consolidated with upfront ASCT at the time of first CR (CR1) after chemoimmunotherapy, patients with *TP53* mutations have significantly reduced 5-year OS or FFS, indicating upfront ASCT cannot overcome the poor prognosis conferred by *TP53* mutations. Since HDT/ASCT hardly provides durable disease control in these high-risk patients and is particularly unfit for chemo-refractory or ASCT ineligible patients, novel treatment strategies are warranted.

CAR T-cell infusion nowadays is becoming the preferred curative option for chemo-refractory or ASCT ineligible patients, or cases that relapse after ASCT. The ZUMA-1, JULIET and TRANSCEND trials indicated ORRs of 41–83% in chemo-refractory patients and ORRs of 53–76% in those with relapse within the first year post-ASCT. Moreover, several clinical trials, including ZUMA-7, BELINDA, and TRANSFORM, are actively assessing whether CAR T-cell therapy could replace salvage HDT/ASCT as second-line treatment in r/r LBCL. Interim analysis of the TRANSFORM study showed the superiority of lisocabtagene maraleucel (Liso-cel) over HDT/ASCT in event-free survival, PFS and CR rate, supporting the potential administration of Liso-cel in earlier lines of therapy in these patients. However, given the genetic heterogeneity of aggressive B-cell lymphomas, it is unclear whether unfavorable genetic aberrations exert uniformly poor outcomes even in the era of adoptive T-cell immunotherapy. Therefore, strategies that can boost the therapeutic effects of CAR T-cell infusion should be actively explored, especially for high-risk cases.

In this study, after CAR19/22 T-cell administration, comparable response and survival data were obtained in patients with or without *TP53* alterations, as week as in patients at different risk levels stratified by four functional classification systems of *TP53* alterations. These findings suggest CAR19/22 T-cell treatment overcame the unfavorable effect of *TP53* disruption in r/r aggressive B-cell lymphoma. We hypothesized that combinational targeting of CD19 and other tumor-associated antigens represents a rational way to cope with *TP53*-disrupted B-cell lymphoma. Recently, a high incidence of CD19-negative relapse provoked by CD19-loss was observed in *TP53*-mutatant B-ALL administered CAR19 T cells^9^. In addition, *TP53* mutation enriched in DLBCL with inconsistent expression of CD19^33,34^. Thus, CD19-negative relapse may be related to irregular CD19 expression in *TP53*-disrupted B-cell lymphoma and driven by CAR19 T-cell surveillance^9,33,34^. Previously, we reported that the CAR19/22 T-cell sequential infusion was highly active and well-tolerated in r/r B-cell malignant cases and could circumvent CD19 antigen escape^31^. Because CD22 and other B-cell markers are usually still expressed on CD19-negative tumor cells^9,35^, combinational targeting of CD19 and other tumor-associated antigens might be a feasible and superior solution than single-targeting of CD19 in *TP53*-disrupted B-cell lymphoma, although validation and investigation of underlying mechanism, including with or without ASCT, in prospective randomized control trials is warranted. Our preliminary underlying mechanism suggests that CAR19 and CAR22 T cells sequentially infused had consistent biological contributions, transcriptional patterns, and clinical relevance with the response or resistance to CAR-T cell therapy *(unpublished data)*. However, the dual CAR targeting strategy includes co-administration or co-transduction of two separate CAR T-cells, T cells transduction using a bicistronic plasmid encoding both CARs, or utilization of a tandem CAR^36^. Novel dual-targeting approaches are being actively examined in preclinical and clinical studies, and the optimal strategy remains undefined and comparatively assessed for clinical benefits^31,36,37^.

Intriguingly, when ASCT was further combined with CAR19/22 T-cell sequential infusion, superior response and survival were obtained in *TP53*-disrupted B-cell lymphoma, even in patients with a SD/PD before transplantation, for whom, as indicated in the SCHOLAR-1 study, ASCT alone can hardly provide durable disease control^1^. In the present study, this combination strategy not only independently predicted improved OS, but also exhibited a reduced occurrence of severe CRS, suggesting potential synergistic effects of ASCT and CAR T-cell therapy. Indeed, multiple studies on metastatic melanoma and B-cell lymphoma have supported the notion that myeloablative ASCT can further augment the effectiveness of adoptive T-cell immunotherapy^38–41^. Myeloablative conditioning before the adoptive transfer of CAR T cells has elevated odds of conferring a beneficial cytokine profile to enhance T cell trafficking or the recruitment of innate immune cells into the microenvironment^42^. In addition, myeloablative conditioning eradicates malignant cells and restrains immunosuppressive elements in the microenvironment that impede the efficiency of CAR T-cell therapy^43–45^. Then, CAR T cells highly activated during hematopoietic reconstitution could further eliminate the residual tumors in both patients and transplants. Finally, reduced tumor load and suppressed regulatory factors, such as monocytes and macrophages, may decrease the rates of CRS and ICANS following CAR T-cell infusion^46,47^. Thus, this novel combination may result in enhanced response, decreased toxicity, reduced relapse, and sustained remission, although further investigation of similarities and differences comparing other dual CAR targeting strategies with or without ASCT is needed. Consistent with long-term outcomes of ZUMA-1 study as well as commercial anti-CD19 CAR T cell therapy^4,48–50^, three months or six months post CAR T-cell infusion were both feasible and efficient timepoints for predicting long-term outcome of CAR-T cell therapy in aggressive B-cell lymphomas. Besides, the present study also highlighted that better ECOG PS had extended survival which was consistent with real-world studies of axicabtagene ciloleucel^51,52^.

Taken together, this is the first clinical study investigating and comparing CAR19/22 T-cell cocktail and its combination with ASCT for effectiveness in *TP53*-disrupted r/r B-cell lymphoma. The results showed CAR19/22 T-cell cocktail was efficacious in this patient population; more importantly, the above findings highlighted great treatment benefits for ASCT combined with CAR T-cell therapy. This study also suggested that, besides *TP53*-disrupted B-cell lymphoma, this combination strategy could be applied for treating aggressive B-cell lymphoma with unfavorable clinicopathological or genetic features. Nevertheless, the present study had several limitations. It was not a randomized control trial, in which the advantages of this combination strategy over either CAR T-cell treatment or ASCT require further validation. In addition, although clinical features were consistent between subgroups, underlying selection bias could hardly be excluded in trials involving transplantation. Finally, varied prognostic relevance of *TP53* alterations may underestimate the potentialities of this combination strategy. In the future, comprehensive genetic studies alongside outcome prediction would help optimize patient selection and achieve individualized treatment in the era of CAR T-cell therapy.

## Materials and methods

### Study design and enrollment

According to the intention-to-treatment principle and the availability of HSCs, r/r B-NHL cases were enrolled in two separate clinical trials examining CAR19/22 T-cell infusion (Trial A) and ASCT incorporating CAR19/22 T-cell therapy (Trial B), respectively. Inclusion and exclusion criteria of Trial A and Trial B are outlined in Supplementary Methods. Both trials had approval from the institutional review board of Tongji Hospital, Tongji Medical College, Huazhong University of Science and Technology, and were registered with the Chinese Clinical Trial Registry (ChiCTR, ChiCTR-OPN-16008526, and 16009847). Signed informed consent was provided by each patient. Both trials were conducted based on the Declaration of Helsinki.

### Clinical procedures

As previously reported^27^, leukapheresis was performed in each patient to collect cells for CAR 19/22 manufacturing. The third-generation CAR employed composed a single-chain variable fragment from a mouse monoclonal antibody targeting human CD19 or CD22, two costimulatory (CD28 and 4–1BB) domains, and the CD3ζ chain as the activation domain. CAR construct validation, as well as cell production and quality control steps, were described previously^41,42^. For patients enrolled in Trial A (CAR19/22 T-cell cocktail infusion), fludarabine (25 mg/m^2^) and cyclophosphamide (300 mg/m^2^) were administered for 3 days (days −4 to −2) with subsequent infusion of CAR19/22 T-cells. CAR19 and CAR22 T cells were administered on successive days from day 0^27^. For Trial B patients (CAR19/22 T-cell cocktail infusion with ASCT), BEAM (300 mg/m^2^ of carmusitine, day −6; 200 mg/m^2^ of etoposide, days −5 to −2; 400 mg/m^2^ of cytarabine, days −5 to −2; 140 mg/m^2^ of melphalan, day −1) at the standard dosage was administered, with subsequent CAR19/22 T-cell infusion (days +2 to +6) following ASCT (day 0). In patients with CAR T following ASCT, CAR19 and CAR22 T cell infusion was carried out within 2–6 days after autologous stem cell infusion^28^. The detailed schematic diagram of study procedures is depicted in Supplemental Fig. 1.

### Laboratory assessments and follow-up

In vivo expansion of CAR19 and CAR22 T cells was assessed by droplet digital polymerase chain reaction (ddPCR) as previously described^53^. Cytokine detection with specific kits was performed as directed by the manufacturer. Cytokine release syndrome (CRS) grading utilized a scale developed by Lee and collaborators. Immune effector cell-associated neurotoxicity syndrome (ICANS) and additional adverse events (AEs) were assessed according to the American Society for Transplantation and Cellular Therapy (ASTCT) Consensus and the National Cancer Institute Common Terminology Criteria for Adverse Events V4.03. Disease stages and treatment responses were examined based on the US National Comprehensive Cancer Network and Lugano Treatment Response Criteria. Follow-up was performed until patient death, loss to follow-up, or consent withdrawal.

### Sequencing and mutational analysis

Panels for targeted sequencing covering the coding sequence (CDS) of the *TP53* gene were designed online (Designstudio Sequencing, Illumina, USA) and shown in Supplemental Table 5. Library preparation was carried out with AmpliSeq^TM^ Library PLUS for Illumina, and sequencing was performed on a NextSeq^TM^ 550 system. Alignment and variant calling utilized the DNA Amplicon workflow (default parameters) on the BaseSpace Sequence Hub. The obtained variants underwent annotation with Annovar. Only exonic nonsynonymous or splice donor/acceptor site variants displaying a frequency below 0.001 in the gnomAD database were filtered for further analyses. The detailed procedures were described previously^23,54^.

### Interphase fluorescence in situ hybridization (FISH)

Interphase FISH was performed to assess formalin-fixed, paraffin-embedded (FFPE) tissue sections or methanol and acetic acid-fixed cells for karyotyping. A commercially available 17p13.1 probe (Vysis, Downers, Grove, IL, USA) was utilized for del(17p) detection.

### Function classification of TP53 alterations

Since distinct prognostic relevance with genetic variants has been demonstrated for the *TP53* gene^55,56^, 4 functional classification systems were utilized to examine whether CAR T-cell treatment could help overcome the unfavorable prognosis resulting from *TP53* gene disruption. In every functional classification system, *TP53* alterations were stratified according to predicted effects on the protein. The first comparison involved missense *TP53* mutations and non-missense mutations, which include nonsense mutations, splice site mutations, small insertions, and deletions^57,58^. Secondly, we classified *TP53* gene mutations as ‘disruptive’ and ‘non-disruptive’ to assess whether the location of a specific *TP53* mutation and the resulting amino acid change had a prognostic value in the current patients, as shown in individuals with AML and solid tumors^59,60^. The definitions of ‘disruptive mutation’ and ‘non-disruptive mutation’ were reported previously^60^. Thirdly, we used the evolutionary action score (EAp53), which performs the functional classification of missense mutations and has been validated in head and neck cancer or AML cases^61^. This system considers evolutionary sensitivity to sequence alteration and amino acid conservation, scoring mutations between 0 (*TP53*wt sequence) and 100. The EAp53 score of a specific *TP53* missense mutation was obtained from the server of the German-Austrian AML Study Group (AMLSG) (http://mammoth.bcm.tmc.edu/EAp53), and a threshold of 75 was utilized to discriminate low-risk (<75) from high-risk (≥75) cases. Finally, we compared patients with or without del(17p).

### Statistical analysis

Comparison of continuous variables utilized the unpaired two-sided student’s *t-*test. Categorical data were assessed by the χ^2^ test or Fisher’s exact test. Differences between ordinal variables were assessed by rank sum analysis. PFS and OS were assessed from the initial infusion to relapse and death, respectively. Duration of response (DOR) was estimated as the time from first response to first radiologically documented progressive disease or death. Follow-up was performed until patient death, loss to follow-up, or consent withdrawal. Objective response rates were presented with 95% confidence intervals (CIs) determined by the Clopper–Pearson method. Survival was assessed by generating Kaplan–Meier curves, and the log-rank test was utilized for comparisons. Univariable and multivariable analyses were carried out with the Cox regression proportional hazards model. Two-sided *P* < 0.05 was deemed statistically significant. SPSS 22.0 (SPSS, Chicago, IL, USA) was utilized for data analysis. The 95%CI of PFS was calculated with the survival (v3.2–11) package of R (v4.0.5; http://www.r-project.org). All data are available from the corresponding authors upon request.

## Supplementary information

Supplemental_Materials

## Data Availability

The additional data collected in this study are available from the corresponding authors on reasonable request.
