# Supplementary material for: Outcome of aggressive B-cell lymphoma with TP53 alterations administered with CAR T-cell cocktail alone or in combination with ASCT
Source: Signal Transduct Target Ther. 2022 Apr 11;7:101. doi: 10.1038/s41392-022-00924-0 (PMC8995369; doi:10.1038/s41392-022-00924-0)
Supplement: Supplementary file 1 — Supplemental_Materials [file 41392_2022_924_MOESM1_ESM.docx]

Supplementary Materials for

Outcome of aggressive B-cell lymphoma with TP53 alteration treated with CAR T-cell cocktail or its combination with ASCT

Jia Wei∗, Min Xiao∗, Zekai Mao∗, Na Wang, Yang Cao, Yi Xiao, Fankai Meng, Weimin Sun, Ying Wang, Xingchen Yang, Liting Chen, Yicheng Zhang, Haichuan Zhu, Shangkun Zhang, Tongcun Zhang, Jianfeng Zhou#, Liang Huang#

Correspondence to: [lhuang@tjh.tjmu.edu.cn](mailto:lhuang@tjh.tjmu.edu.%20cn) or [jfzhou@tjh.tjmu.edu.cn](mailto:jfzhou@tjh.tjmu.edu.cn)

**This PDF file includes:**

Materials and Methods

Figures. S1 to S13

Tables S1 to S5

**Materials and Methods**

Inclusion and exclusion criteria

**1. Trial A:** An open-label, single-center and single-arm clinical study for the sequential infusion of anti-CD22 and anti-CD19 CAR T-cell cocktail (CAR19/22 T-cell cocktail) for patients with relapsed or refractory (r/r) B-cell malignancies (ChiCTR, number ChiCTR-OPN-16008526).

**Inclusion criteria:**

Subjects must meet all the following criteria to be enrolled in this study:

1. Signed written informed consent obtained prior to any study procedures.
2. Age ≥ 18 years at the time of consent.
3. Subjects with CD19^+^ and CD22^+^ B-cell acute lymphoblastic leukemia (B-ALL) or B-cell non-Hodgkin lymphoma (B-NHL) diagnosed by pathology and histology.
4. Relapsed or refractory B-cell malignancy of the following histology: B-ALL; Aggressive B-cell lymphoma, including diffuse large B-cell lymphoma (DLBCL), Burkitt’s lymphoma (BL), mantle cell lymphoma (MCL) and transformed B-cell lymphoma.
5. In one of the following conditions:
   1. With relapsed or refractory disease after 2nd-line salvage therapy.
   2. With relapsed or refractory disease after hematopoietic stem cell transplant (HSCT).
   3. With relapsed or refractory disease and high-risk clinical or pathological features, including double/triple-hit lymphoma, et al.
   4. With B-ALL, but ineligible for allogenic HSCT.
   5. With B-NHL, but ineligible for autologous HSCT.
6. Subjects with measurable lesions.
7. Adequate organ function, defined as creatinine<2.5mg/dl; aspartate transaminase/ alanine transaminase <3 × upper limit of normal; SiO2 ≥ 95%; bilirubin < 2.0 mg/dl; LVEF > 40%.
8. Adequate vascular access for leukapheresis procedure.
9. Eastern Cooperative Oncology Group (ECOG) performance status of ≤ 2.
10. Estimated survival of ≥ 3 months.

**Exclusion criteria：**

Subjects who meet any of the following criteria will be excluded from participation in this study:

1. Pregnant or nursing women.
2. Planning pregnancy within 1 year.
3. Active hepatitis B, hepatitis C, or human immunodeficiency virus (HIV) infection at the time of screening.
4. Systemic fungal, bacterial, viral, or other infection that is not controlled, at the time of screening.
5. History of accepting systemic steroids treatments within 4 weeks.
6. Allergic to any cytokines or antibodies.
7. Participation in an investigational research study within 6 weeks before enrolled.
8. Present active graft-versus host disease.
9. History of mental disorders.
10. History of another primary malignancy.
11. Drug abuse and addiction.
12. Other conditions deemed inappropriate by the investigator for enrollment.

**2. Trial B:** An open-label, single center, single arm clinical study for the sequential infusion of anti-CD19 CAR-T and anti-CD22 CAR-T therapy following autologous hematopoietic stem cell transplantation (ASCT) for relapsed, refractory, and high-risk B cell lymphoma (ChiCTR, number ChiCTR-OPN-16009847).

**Inclusion criteria:**

Subjects must meet all the following criteria to be enrolled in this study:

1. Signed written informed consent obtained prior to any study procedures.
2. Age ≥ 18 years at the time of consent.
3. Subjects with CD19^+^ and CD22^+^ B-cell acute lymphoblastic leukemia (B-ALL) or B-cell non-Hodgkin lymphoma (B-NHL) diagnosed by pathology and histology.
4. Relapsed or refractory B-cell malignancy of the following histology: B-ALL; Aggressive B-cell lymphoma, including diffuse large B-cell lymphoma (DLBCL), Burkitt’s lymphoma (BL), mantle cell lymphoma (MCL) and transformed B-cell lymphoma.
5. In one of the following conditions:
   1. With relapsed or refractory disease after 2nd-line salvage therapy.
   2. With relapsed or refractory disease after hematopoietic stem cell transplant.
   3. With relapsed or refractory disease and high-risk clinical or pathological features, including double/triple-hit lymphoma, et al.
   4. With B-ALL, but ineligible for allogenic HSCT.
6. Subjects with measurable lesions.
7. Adequate organ function, defined as creatinine<2.5mg/dl; aspartate transaminase/ alanine transaminase <3 × upper limit of normal; SiO2 ≥ 95%; bilirubin < 2.0 mg/dl; LVEF > 40%.
8. Adequate vascular access for leukapheresis procedure.
9. Eastern Cooperative Oncology Group (ECOG) performance status of ≤ 2.
10. Estimated survival of ≥ 3 months.

**Exclusion criteria:**

Subjects who meet any of the following criteria will be excluded from participation in this study:

1. Pregnant or nursing women.
2. Planning pregnancy within 1 year.
3. Active hepatitis B, hepatitis C, or human immunodeficiency virus (HIV) infection at the time of screening.
4. Systemic fungal, bacterial, viral, or other infection that is not controlled, at the time of screening.
5. History of accepting systemic steroids treatments within 4 weeks.
6. Allergic to any cytokines or antibodies.
7. Participation in an investigational research study within 6 weeks before enrolled.
8. Present active graft-versus host disease.
9. History of mental disorders.
10. History of another primary malignancy.
11. Drug abuse and addiction.
12. Other conditions deemed inappropriate by the investigator for enrollment.

**Supplemental figures**

**
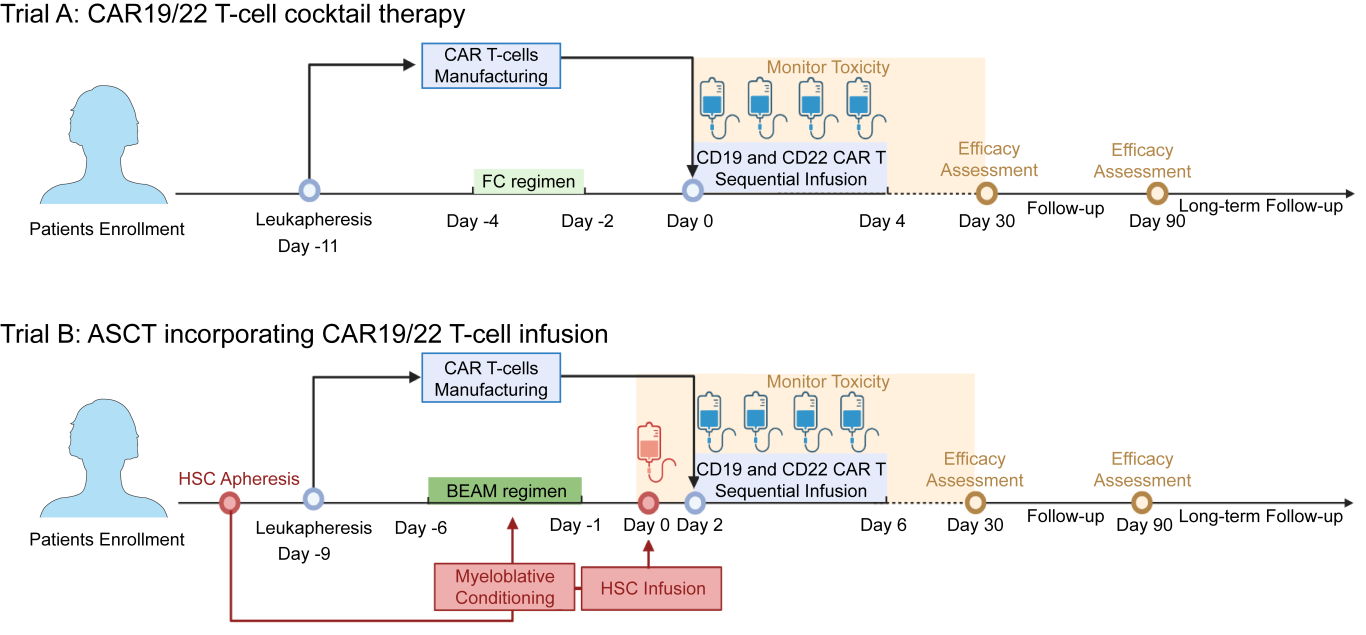
Figure. S1****.**

**Figure. S1. Schematic diagram of study procedures.** Patients in trial A were given fludarabine and cyclophosphamide for 3 days (FC regimen) as lymphodepletion chemotherapy and subjected to the sequential infusion of CAR19/22 T-cell cocktail. CAR19 and CAR22 T cells were infused separately on successive days from day zero as reported before^1^. Patients in trial B were given bis-carmusitine, etoposide, cytarabine and melphalan (BEAM regimen) as myeloablative chemotherapy and received the infusion of autologous stem cells on day 0 and CAR19/22 T-cell cocktail on the following days (from +2 to +6)^2^.

**Figure. S2****.**

**
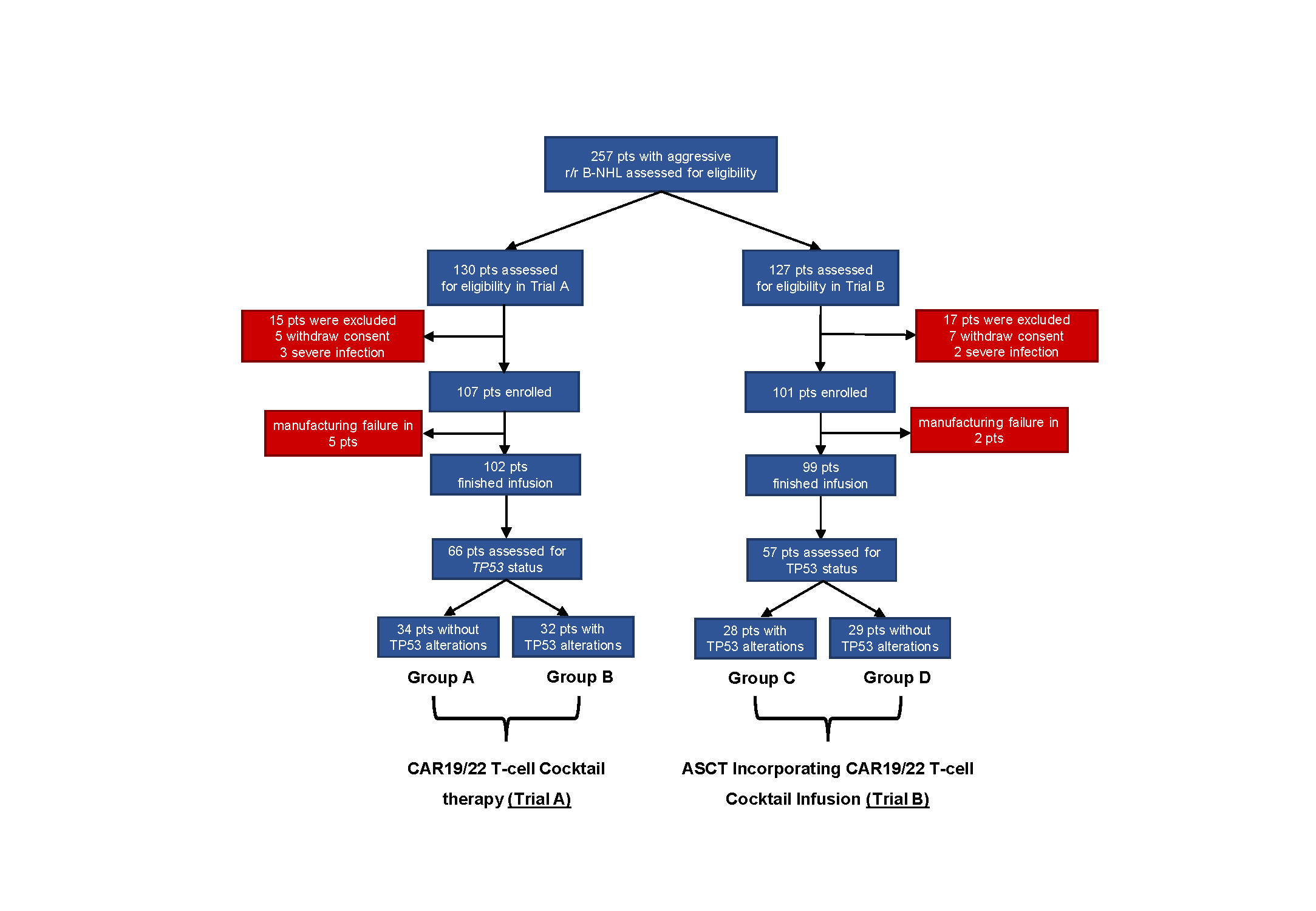
**

**Figure. S2. Flowchart of patient enrollment and group assignment.** From September 2016 through September 2020, 257 patients with r/r aggressive B-NHL were screened for the eligibility of these 2 trails and 208 patients finally enrolled. 102 patients in Trail A and 99 patients in Trial B finished CAR T-cell infusion. TP53 mutation and del(17p) were screened in 66 patients in Trial A and 57 patients in Trial B. In Trial A, 32 patients (48.5%, group B) carried *TP53* alterations, while 34 patients (51.5%, group A) did not. One patient in Group B who had grade 5 infection and died of septic shock within the first month were excluded from response evaluation and survival assessment but were included in toxicity assessment as specified in the protocol. In trial B, 28 patients (49.1%, group C) carried *TP53* alterations, while 29 patients (50.9%, group D) did not. The cutoff date for data collection was April 30, 2021. pts, patients.

**
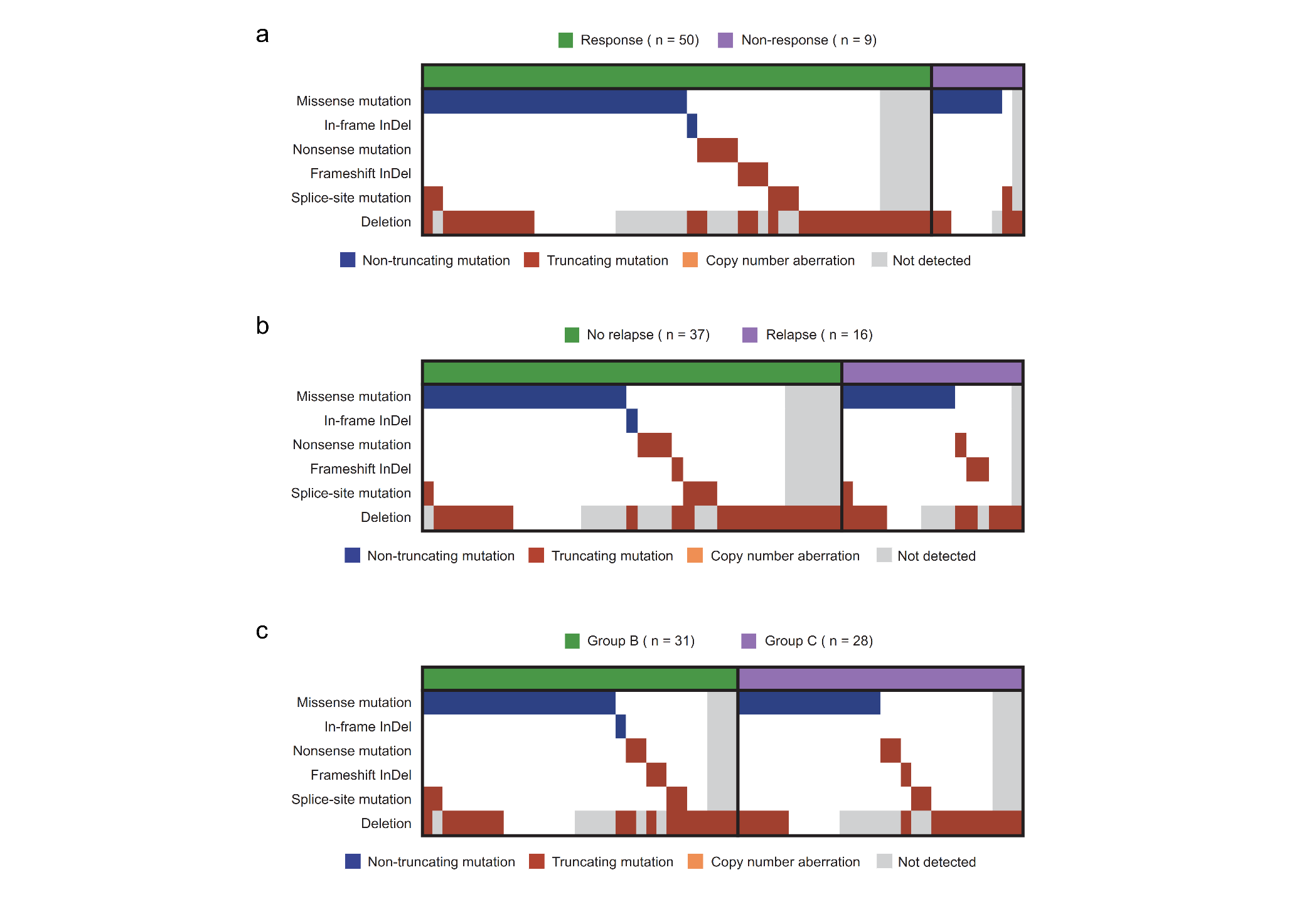
Figure. S3.**

**Figure. S3. Genetic aberrations identified in patients treated with CAR T-cell therapy.** Types of TP53 aberration are shown as rows, and samples are shown as columns. a. Mutation patterns in responders and non-responders. b. Mutation patterns in relapse patients and other patients. c. Mutation patterns in patients of Group B and Group C.

**
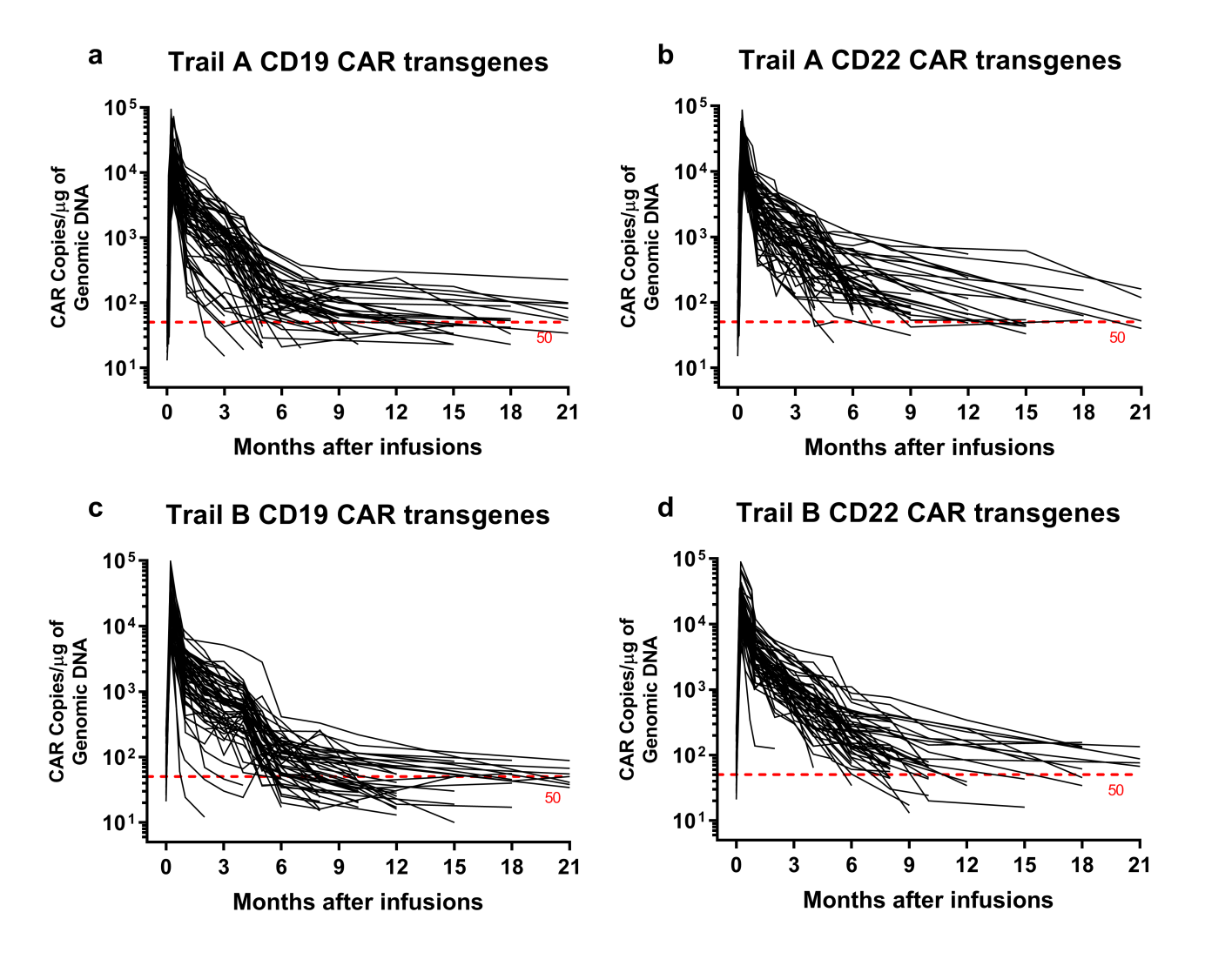
Figure. S4.**

**Figure. S4. Cellular kinetics of CAR19 and CAR22 transgenes in peripheral blood**. The dotted red lines in each panel denote the lower limit of quantitation (50 copies/µg). **a** and **b**. Copies of CD19 and CD22 CAR transgenes, respectively, in patients enrolled in Trial A. **c** and **d**. Copies of CD19 and CD22 CAR transgenes, respectively, in patients enrolled in Trial B.

**Figure. S5.**

**
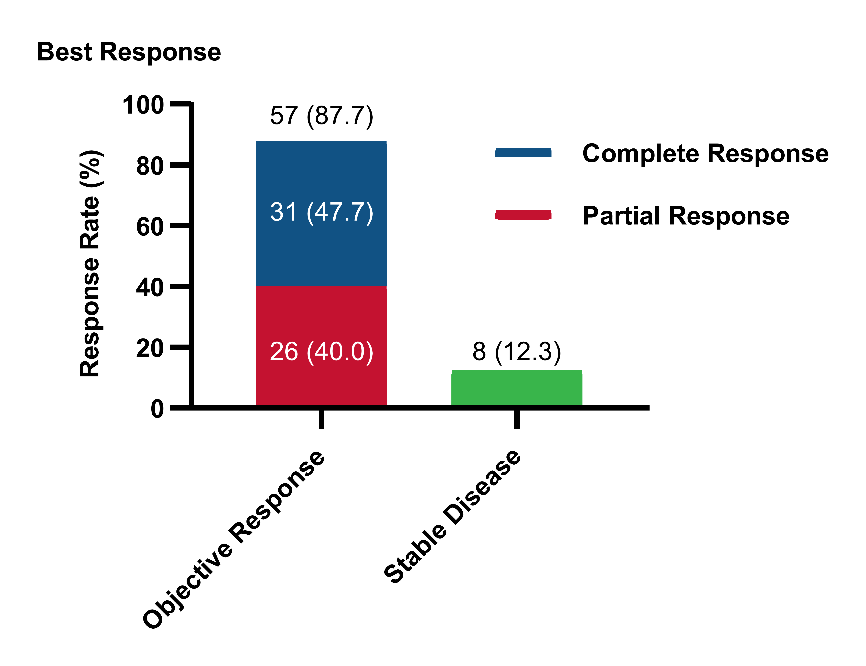
**

**Figure. S5. Best response achieved in Trial A.** Numbers and percentages (in brackets) are shown for r/r aggressive B-NHL patients who had an objective response (OR, complete response and partial response, CR+PR) or stable disease (SD), respectively, when treated with CAR19/22 T-cell cocktail therapy in Trial A.

**
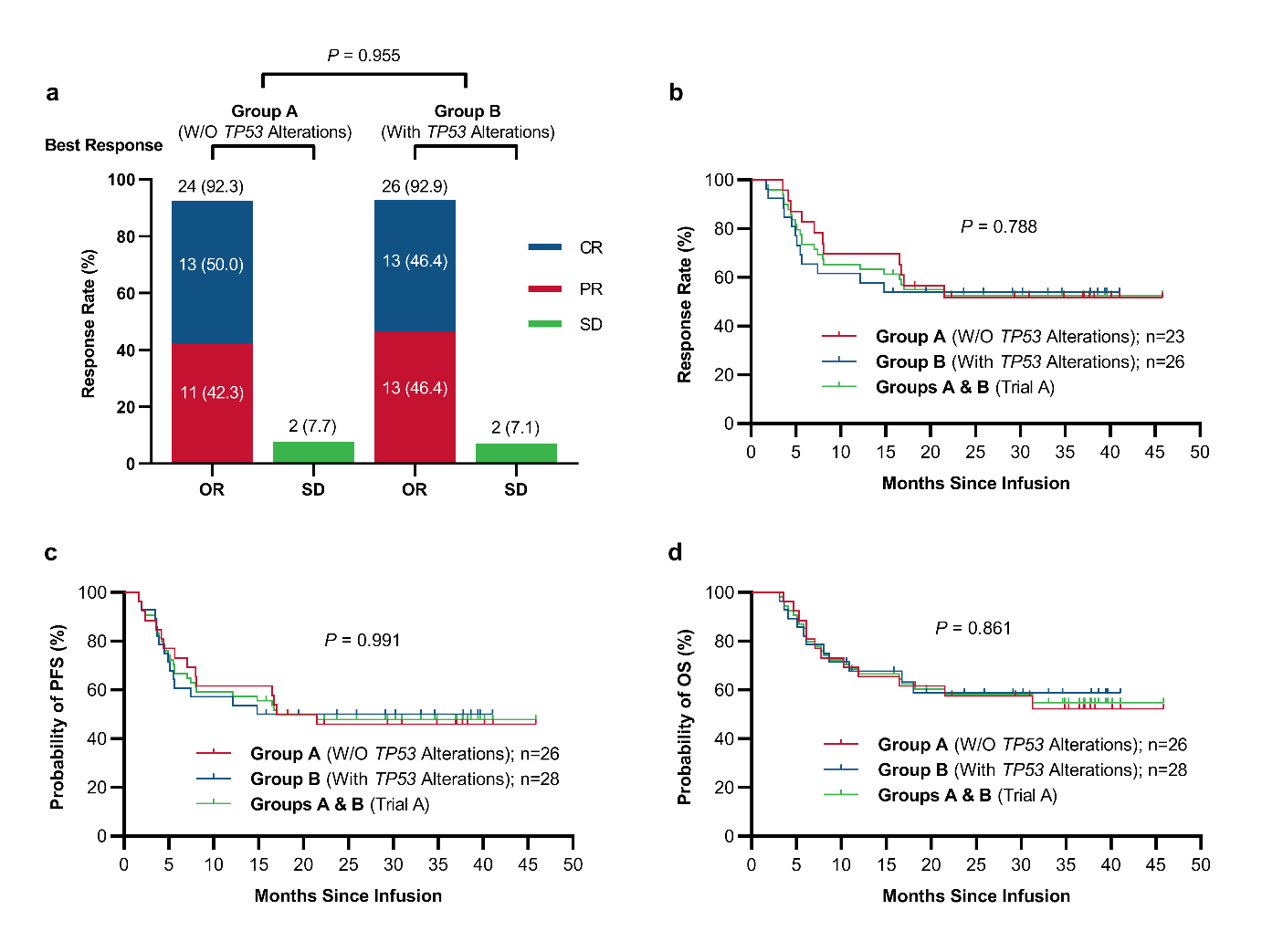
Figure. S6.**

**Figure. S6. Similar outcome in r/r DLBCL patients when treated with CAR19/22 T-cell cocktail, irrespective of *TP53* alteration status.** **a.** The comparison of best response in the cohort of r/r DLBCL between patients with or without *TP53* alterations. **b.** The Kaplan-Meier estimate of DOR in the cohort of r/r DLBCL between patients with or without *TP53* alterations. **c.** The Kaplan-Meier estimate of PFS in the cohort of r/r DLBCL between patients with or without *TP53* alterations. **d.** The Kaplan-Meier estimate of OS in the cohort of r/r DLBCL between patients with or without *TP53* alterations. ORR, objective response rate; OR, objective response; CR, complete response; PR, partial response; SD, stable disease; DOR, duration of response; W/O, without.

**Figure. S7.**

**
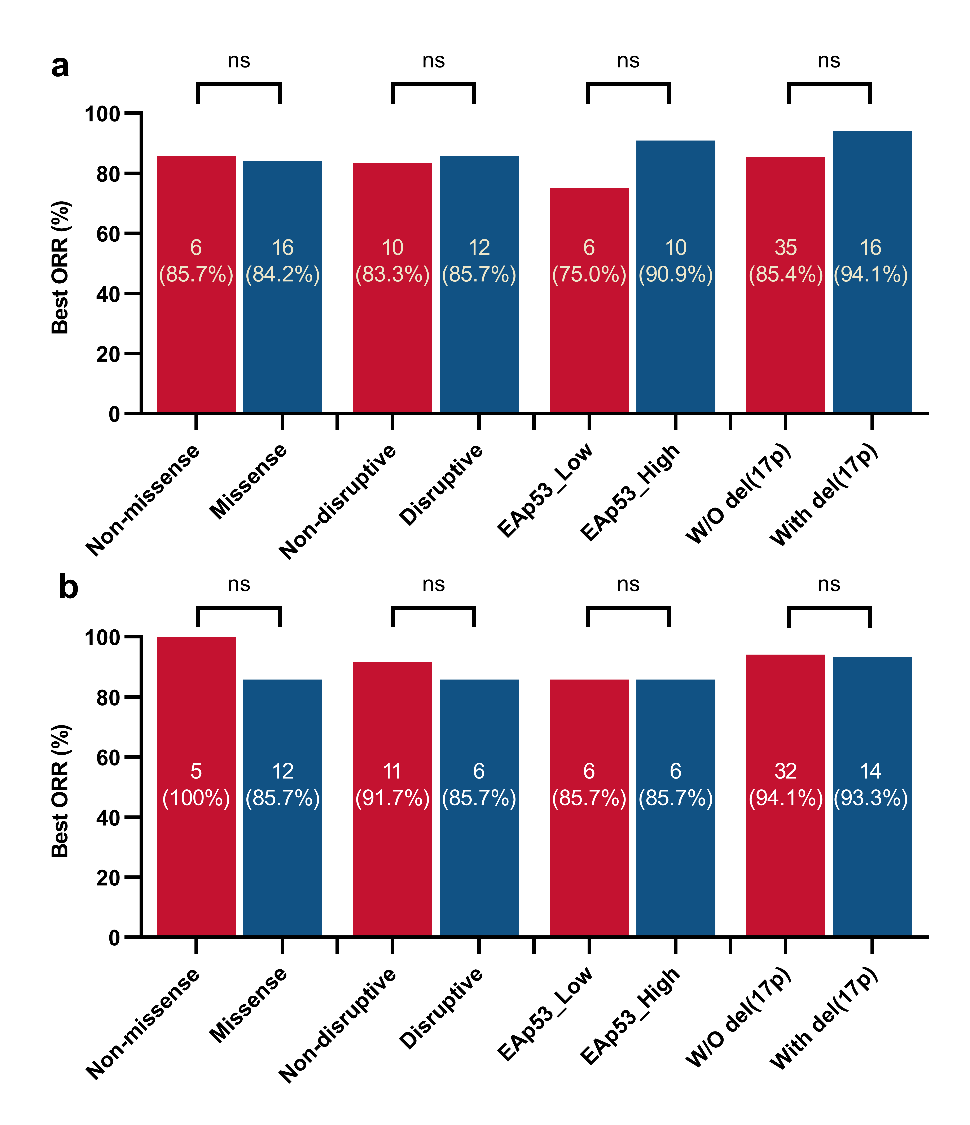
**

**Figure. S7.** **Similar best ORR of patients stratified by 4 *TP53* functional classification systems when treated with CAR19/22 T-cell cocktail or ASCT incorporating CAR19/22 T-cell cocktail infusion.** This diagram shows ORR of patients who had r/r aggressive B-NHL and concurrent *TP53* alterations, when treated with CAR19/22 T-cell cocktail in group B (a) and ASCT incorporating CAR19/22 T-cell cocktail infusion in group C (b). Since prognostic relevance varies according to distinct *TP53* alterations, 4 functional classification systems are applied to further elucidate whether CAR T-cell cocktail therapy can overcome the unfavorable prognostic impact conferred from *TP53* alterations. ORR, objective response rate; ns, no significance, analyzed by Fisher exact tests; W/O, without.

**Figure. S8.**

**
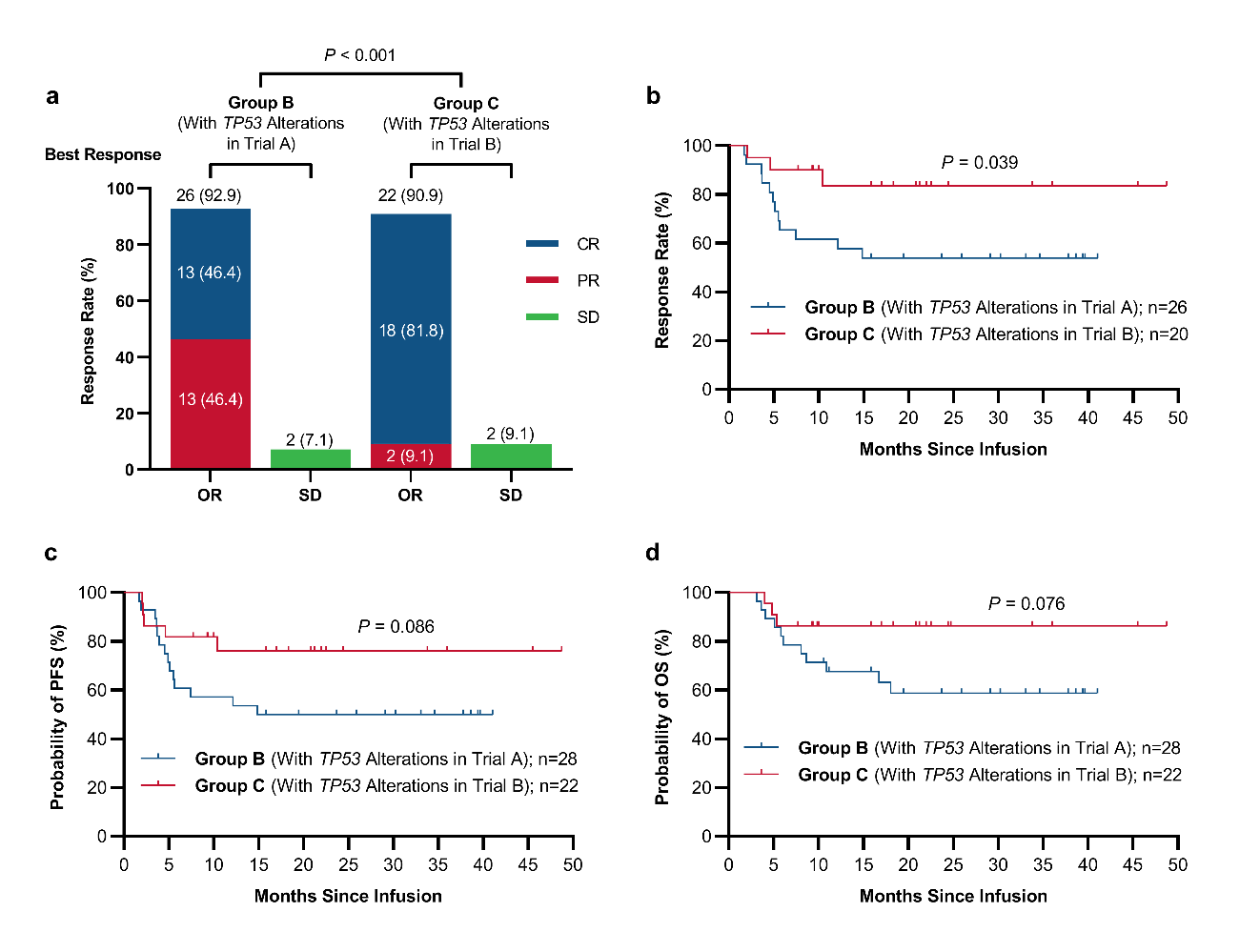
**

**Figure. S8. Superior outcome** **in patients with r/r DLBCL and concurrent *TP53* alterations when treated with ASCT incorporating CAR19/22 T-cell cocktail infusion.** **a.** The comparison of best response when treated with CAR19/22 T-cell cocktail infusion alone or incorporated in ASCT. When treated with ASCT incorporating CAR19/22 T-cell cocktail infusion, the best ORR was 90.9% (95% CI: 72.2% - 98.4%), with a best CR of 81.8% (95% CI: 61.5% - 92.7%). **b-d.** The Kaplan-Meier estimate of DOR (B), PFS (C) and OS (D) when treated with CAR19/22 T-cell cocktail infusion alone or incorporated in ASCT. With a median follow-up of 18.3 (range: 4.0- 48.7) months, the median DOR, PFS and OS was not reached. The estimated 24-month PFS and OS rates were 76.0% (95% CI: 51.2% - 89.3%) and 86.4% (95% CI: 63.4% - 95.4%), respectively. ORR, objective response rate; OR, objective response; CR, complete response; PR, partial response; SD, stable disease; DOR, duration of response.

**Figure. S9.**

**
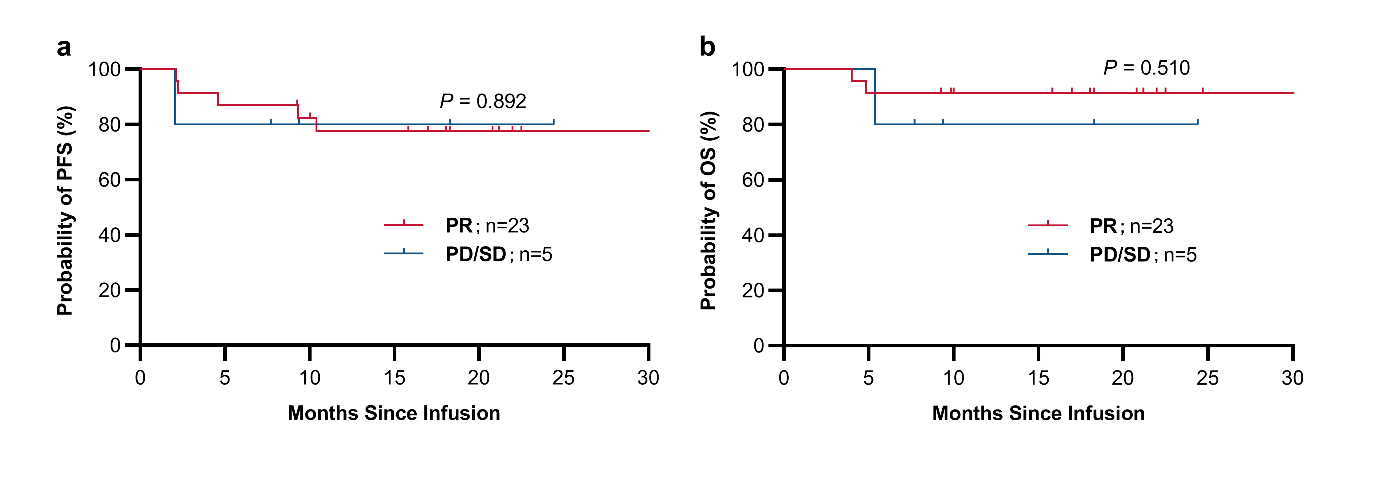
**

**Figure. S9.** **Outcomes in r/r aggressive B-cell lymphoma with concurrent *TP53* alterations according to the response at enrollment when CAR19/22 T-cell cocktail infusion incorporated in ASCT.** The Kaplan-Meier estimates of PFS (a) or OS (b) according to response at enrollment.


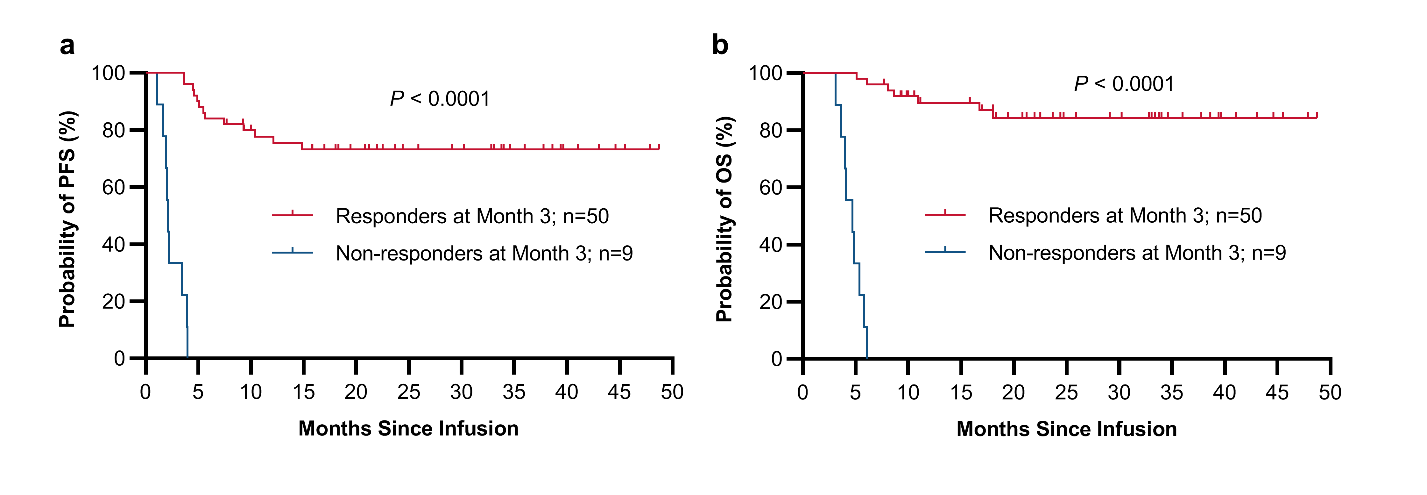

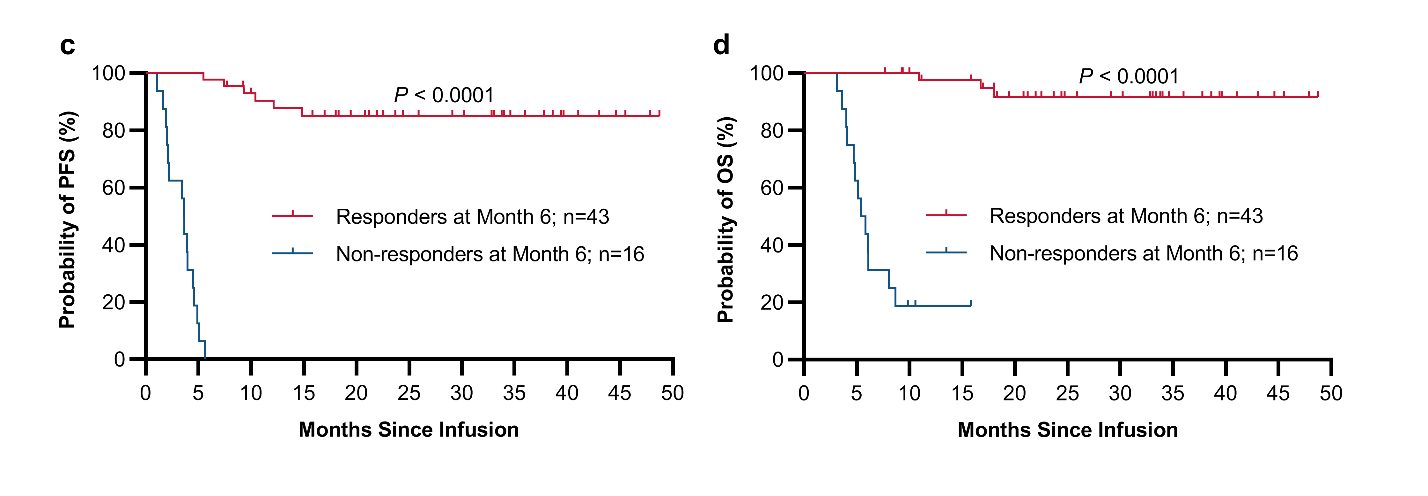
**Figure. S10.**

**Figure. S10. R3m predicts the long-term outcome of patients with r/r aggressive B-NHL and concurrent *TP53* alterations.** Among the patients with *TP53* alterations, patients who maintained response at month 3 (R3m, defined as “durable complete or partial response at month 3) (responders) had significantly extended PFS and OS when compared with patients who failed to have R3m (non-responders). **a.** The Kaplan-Meier estimate of PFS for responders and non-responders. **b.** The Kaplan-Meier estimate of OS for responders and non-responders. The Kaplan-Meier estimates of PFS (**c**) or OS (**d**) for responders and non-responders at month 6. With a median follow-up of 21.6 (range: 5.1 - 48.7) months, the estimated 24-month PFS and OS rates in responders were 73.2% (95% CI: 58.3% - 83.5%) and 84.3% (95% CI: 69.7% - 92.3%), respectively.

**Figure. S11.**

**
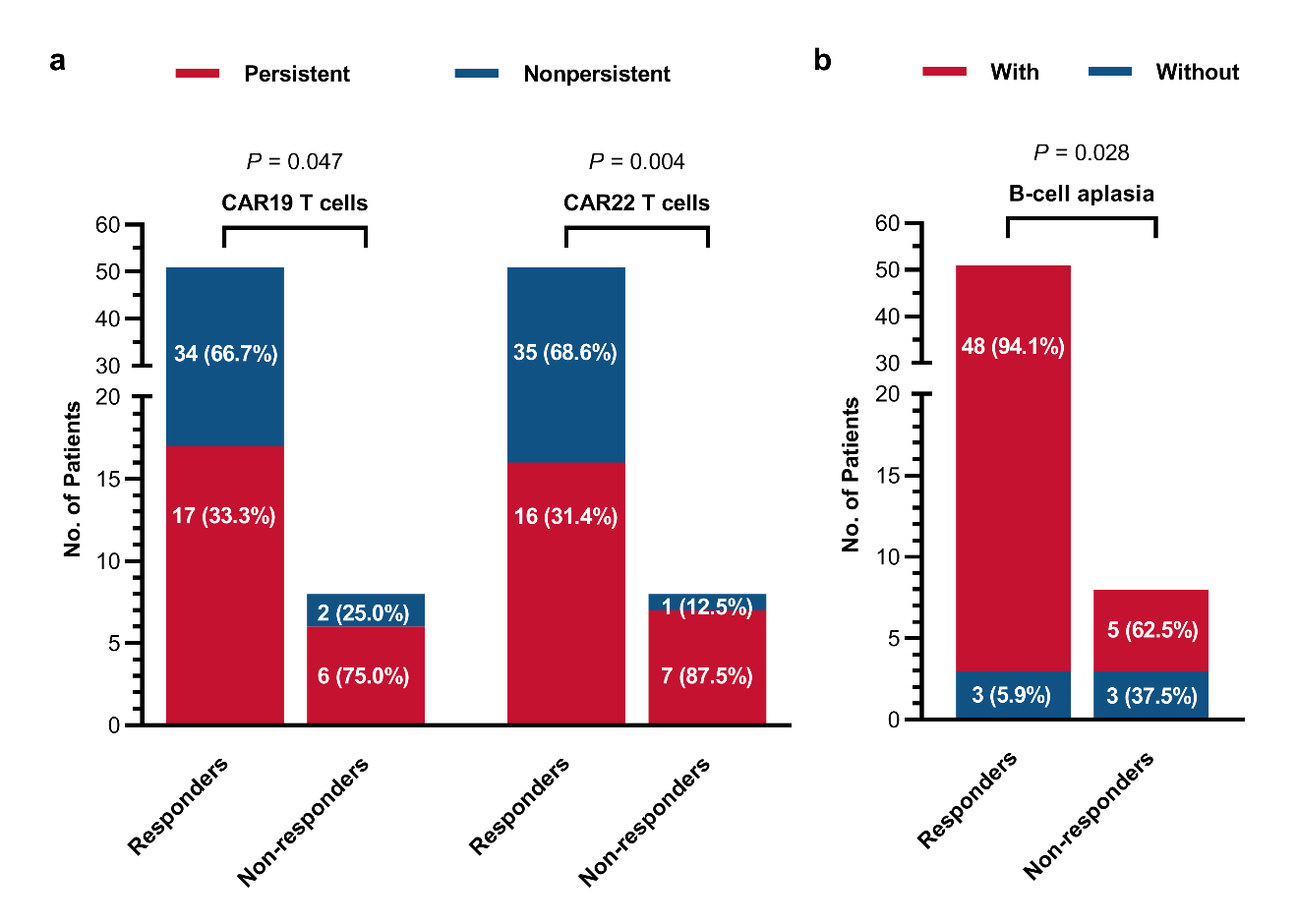
**

**Figure. S11. Responders had more persistent CAR transgenes and longer lasting B-cell aplasia. a.** Comparison of CAR19 and CAR22 persistence between responders and non-responders at month 3. Genomic DNA was isolated from the samples of whole blood at serial time points before and after the infusion of CAR T cells. Lentiviral copies of CAR19 or CAR22 transgenes were assessed by droplet digital polymerase chain reaction. The existence of CAR was defined as greater than 50 copies/ug DNA^3^. **b.** Comparison of B-cell aplasia between responders and non-responders at month 3. B-cell aplasia is defined as <1% B cells/ WBCs or < 3% B cells/lymphocytes (the lower limit of normal for blood B-cell levels).

**Figure. S12.**

**
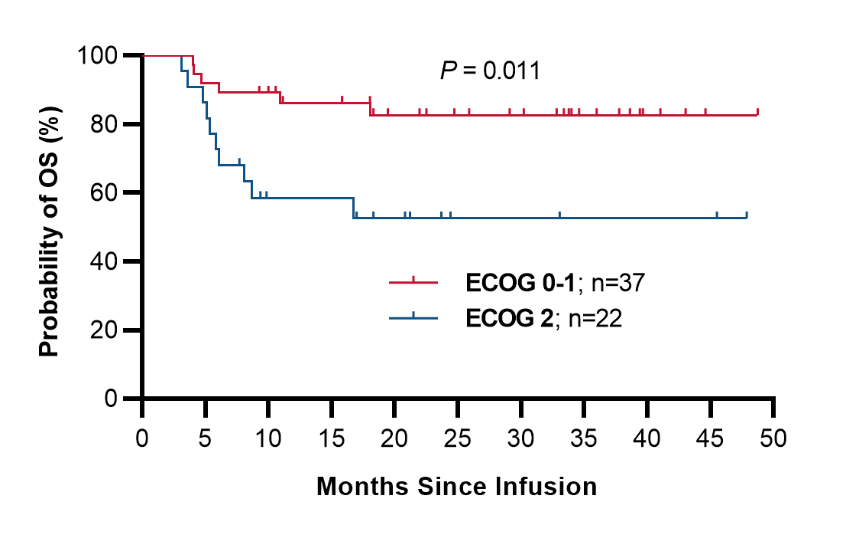
**

**Figure. S12. Better ECOG PS predicts improved OS in patients with r/r aggressive B-NHL and concurrent *TP53* alterations.** In Log-rank survival analysis, OS was significantly improved in patients who had superior performance status (ECOG PS 0-1) than in patients who had inferior performance status (ECOG PS 2) (*P* = .011). ECOG, Eastern Cooperative Oncology Group; PS, performance status.

**Figure. S13.**

**
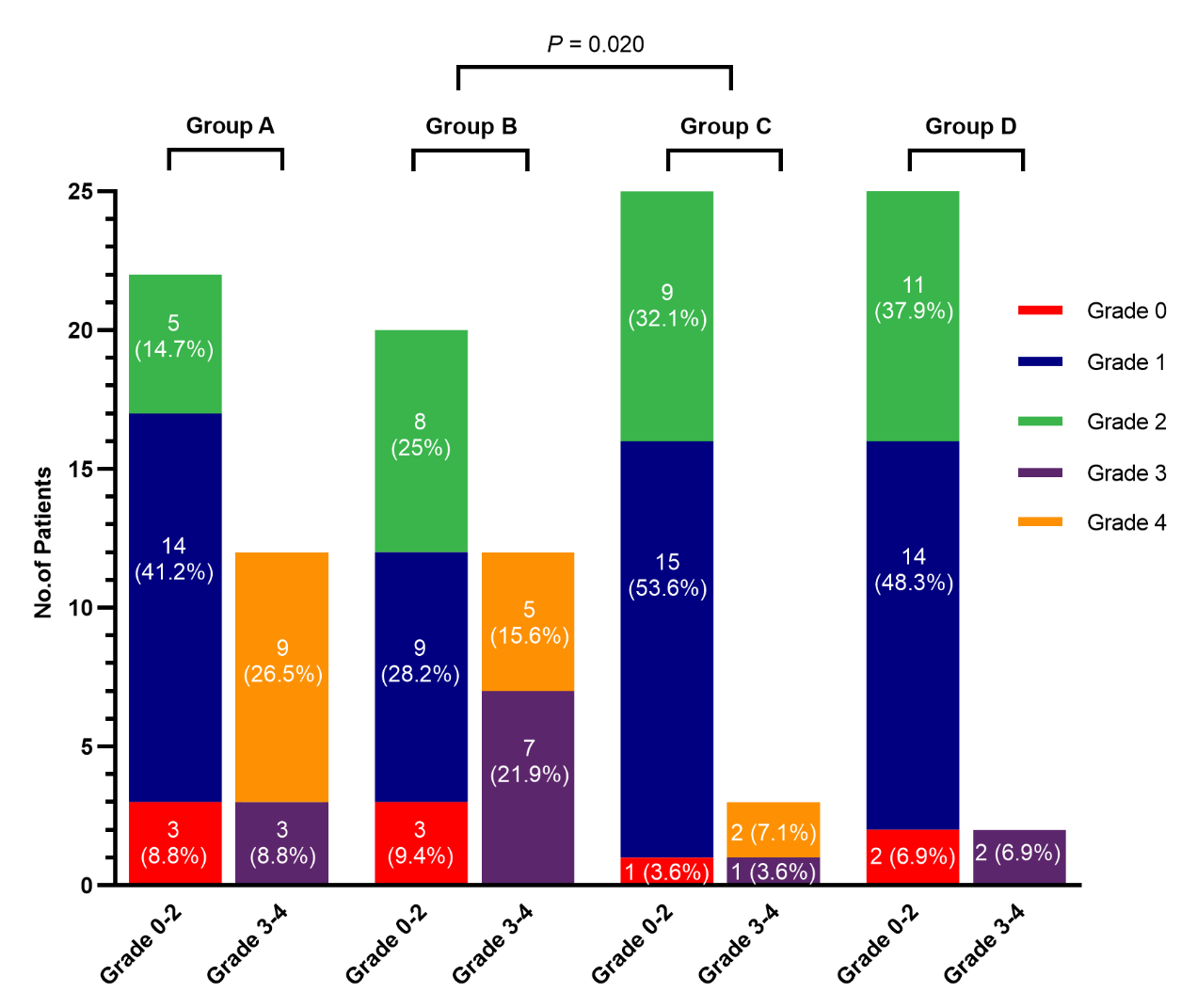
**

**Figure. S13. Grading and comparison of CRS in each group.** Among the patients with r/r B-NHL and concurrent *TP53* alterations, patients who undergone ASCT incorporating CAR19/22 T-cell infusion (group C in Trial B) had lower incidence (10.7% vs. 37.5%, *P* = .020) of severe CRS (grade ≥ 3) than patients who received CAR19/22 T-cell therapy (group B in Trial A). CRS, cytokine release syndrome; *P*-value was calculated by Fisher exact tests.

**Supplemental tables**

**Table S1. ORR of R3m with different *TP53* alteration pattern**

| **Subgroups** | **No. of pts** | **No. of pts with OR at M3** | **Percentage of pts with OR （95% CI)** | | ***P* value** |
| --- | --- | --- | --- | --- | --- |
| 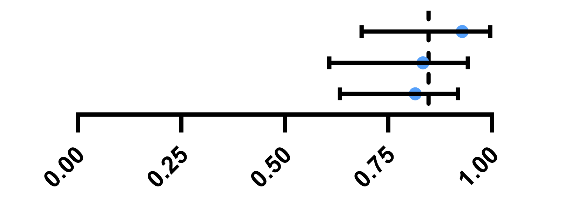**TP53 alteration pattern** | | | | | 0.618 |
| TP53 mutation | 27 | 22 |  | 0.8148(0.6330-0.9182) |  |
| TP53 mut & del | 18 | 15 |  | 0.8333(0.6078-0.9416) |  |
| *TP53* deletion | 14 | 13 |  | 0.9286(0.6853-0.9963) |  |

**Table S2. Subgroup analysis of R3m in Group B**

| **Subgroups** | **No. of pts** | 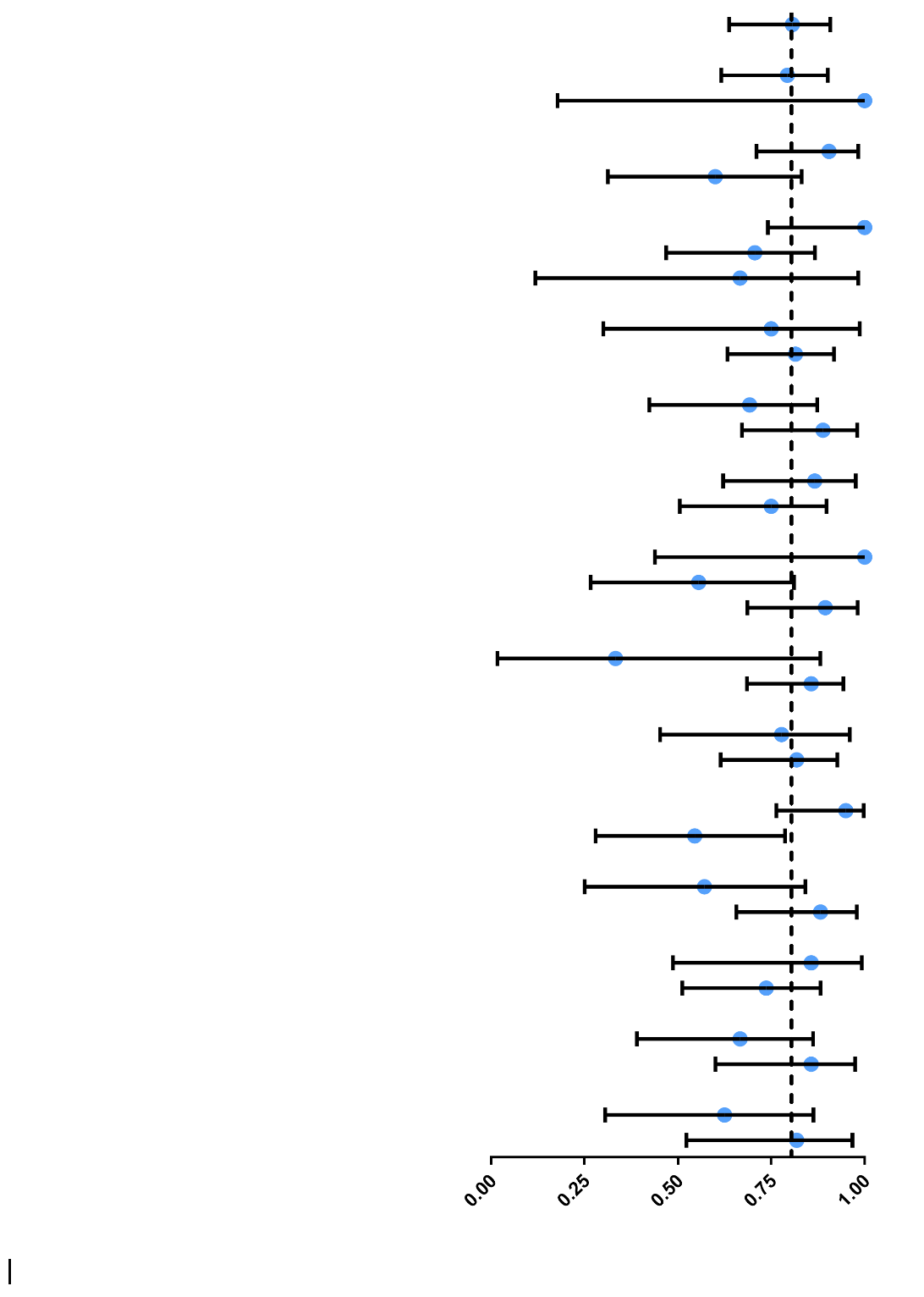**No. of pts with OR at M3** | **Percentage of pts with OR (95% CI)** | | ***P* value** |
| --- | --- | --- | --- | --- | --- |
| **Overall** | 31 | 25 |  | 0.806(0.637-0.908) |  |
| **Age** | | | | | >0.999 |
| **<60** | 29 | 23 |  | 0.793(0.616-0.902) |  |
| **≥60** | 2 | 2 |  | 1.0 (0.178-1.0) |  |
| **ECOG PS** |  |  |  |  | 0.067 |
| **0-1** | 21 | 19 |  | 0.905(0.711-0.983) |  |
| **2** | 10 | 6 |  | 0.6(0.313-0.932) |  |
| **Baseline LDH ratio** | | | | | 0.128 |
| **<1×ULN** | 11 | 11 |  | 1.0(0.741-1.0) |  |
| **1-3×ULN** | 17 | 12 |  | 0.706(0.490-0.867) |  |
| **>3×ULN** | 3 | 2 |  | 0.667(0.118-0.983) |  |
| **Disease stage** | | | | | >0.999 |
| **I or II** | 4 | 3 |  | 0.75(0.301-0.987) |  |
| **III or IV** | 27 | 22 |  | 0.815(0.633-0.918) |  |
| **IPI** | | | | | 0.208 |
| **0-2** | 13 | 9 |  | 0.692(0.424-0.873) |  |
| **3-5** | 18 | 16 |  | 0.889(0.672-0.980) |  |
| **Tumor mass** |  |  |  |  | 0.654 |
| **<5cm** | 15 | 13 |  | 0.867(0.621-0.976) |  |
| **≥5cm** | 16 | 12 |  | 0.75(0.505-0.898) |  |
| **No. of prior-treatment lines** | | | |  | 0.071 |
| **2** | 3 | 3 |  | 1.0(0.438-1.0) |  |
| **3** | 9 | 5 |  | 0.556(0.267-0.811) |  |
| **≥4** | 19 | 17 |  | 0.895(0.686-0.981) |  |
| **Previous ASCT** | | | |  | 0.088 |
| **Yes** | 3 | 1 |  | 0.3333(0.0171-0.8815) |  |
| **No** | 28 | 24 |  | 0.8571 (0.6851-0.9430) |  |
| **Bridging treatment** |  |  |  |  | >0.999 |
| **Yes** | 9 | 7 |  | 0.778(0.453-0.960) |  |
| **No** | 22 | 18 |  | 0.818 (0.615-0.927) |  |
| **CRS** |  |  |  |  | 0.013* |
| **0-2** | 20 | 19 |  | 0.95(0.764-0.997) |  |
| **≥3** | 11 | 6 |  | 0.545(0.280-0.787) |  |
| **Del(17p)** (n=24) | | | | | 0.127 |
| **Without** | 7 | 4 |  | 0.571(0.250-0.842) |  |
| **With** | 17 | 15 |  | 0.882(0.657-0.979) |  |
| ***TP53*mut Functional classification Ⅰ** (n=26) | | | | | >0.999 |
| **Non-missense** | 7 | 6 |  | 0.857(0.487-0.993) |  |
| **Missense** | 19 | 14 |  | 0.737(0.512-0.882) |  |
| ***TP53*mut Functional classification ⅠI** (n=26) | | | |  | 0.365 |
| **Non-disruptive** | 12 | 8 |  | 0.667(0.391-0.862) |  |
| **Disruptive** | 14 | 12 |  | 0.857(0.601-0.975) |  |
| ***TP53*mut Functional classification ⅠII** (n=19) | | | |  | 0.603 |
| **Low EAp53** | 8 | 5 |  | 0.625(0.306-0.863) |  |
| **High EAp53** | 11 | 9 |  | 0.818(0.523-0.968) |  |

**Table S3. Subgroup analysis of R3m in Group C**

| **Subgroups** | **No. of pts** | 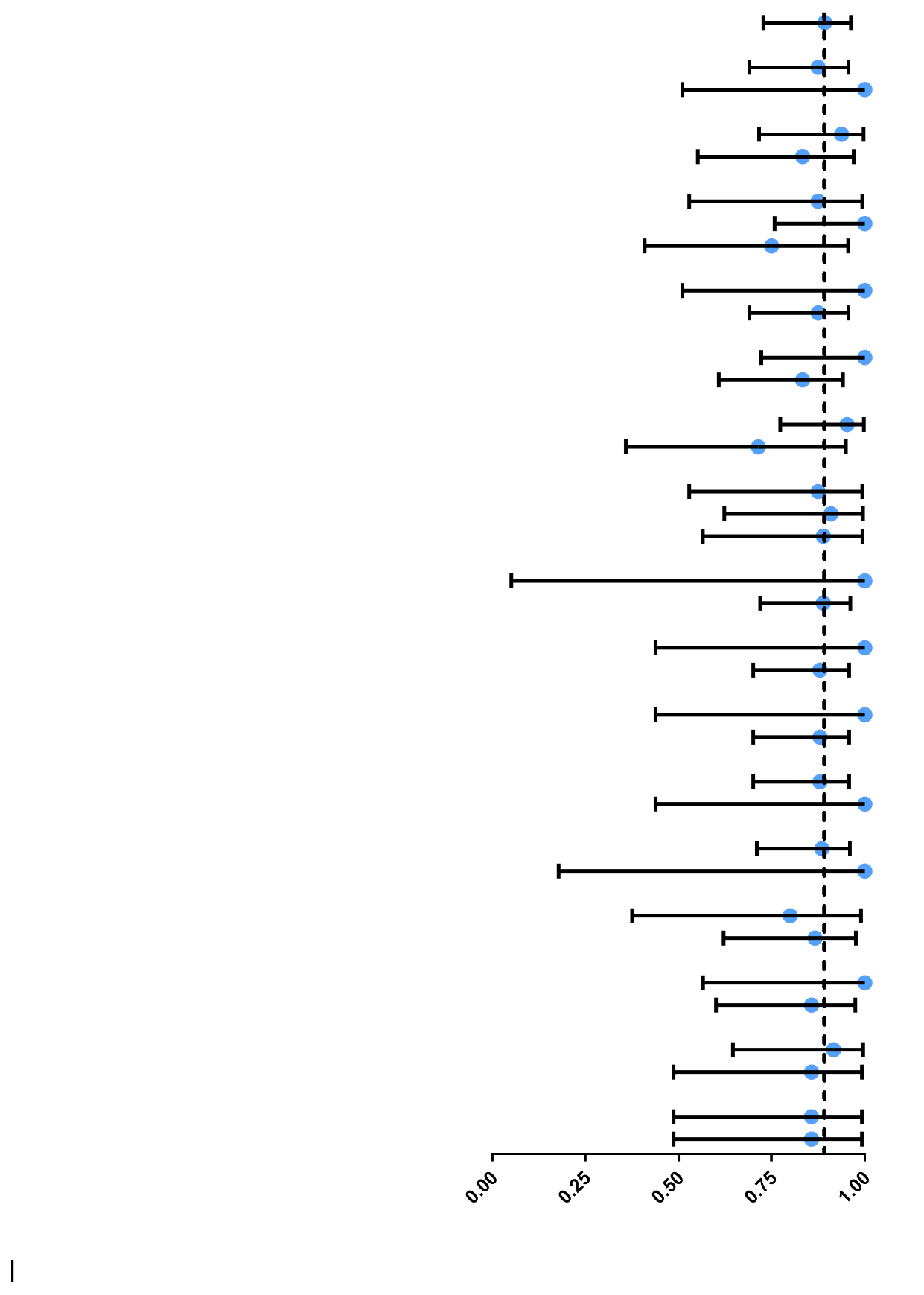**No. of pts with OR at M3** | **Percentage of pts with OR (95% CI)** | | ***P* value** |
| --- | --- | --- | --- | --- | --- |
| **Overall** | 28 | 25 |  | 0.893(0.728-0.963) |  |
| **Age** | | | | | >0.999 |
| **<60** | 24 | 21 |  | 0.875(0.690-0.957) |  |
| **≥60** | 4 | 4 |  | 1.0(0.510-1.0) |  |
| **ECOG PS** |  |  |  |  | 0.560 |
| **0-1** | 16 | 15 |  | 0.938(0.717-0.997) |  |
| **2** | 12 | 10 |  | 0.833 (0.552-0.970) |  |
| **Baseline LDH ratio** | | | | | 0.205 |
| **<1×ULN** | 8 | 7 |  | 0.875(0.529-0.994) |  |
| **1-3×ULN** | 13 | 11 |  | 0.846(0.578-0.973) |  |
| **>3×ULN** | 8 | 6 |  | 0.75(0.409-0.956) |  |
| **Disease stage** | | | | | >0.999 |
| **I or II** | 4 | 4 |  | 1.0(0.5101-1.0) |  |
| **III or IV** | 24 | 21 |  | 0.875(0.6900-0.9866) |  |
| **IPI** | | | | | 0.533 |
| **0-2** | 10 | 10 |  | 1.0(0.722-1.0) |  |
| **3-5** | 18 | 15 |  | 0.833(0.608-0.942) |  |
| **Tumor mass** |  |  |  |  | 0.145 |
| **<5cm** | 21 | 20 |  | 0.952(0.773-0.998) |  |
| **≥5cm** | 7 | 5 |  | 0.714(0.359-0.949) |  |
| **No. of prior-treatment lines** | | | |  | 0.971 |
| **2** | 8 | 7 |  | 0.875 (0.529-0.994) |  |
| **3** | 11 | 10 |  | 0.909 (0.623-0.995) |  |
| **≥4** | 9 | 8 |  | 0.889(0.565-0.994) |  |
| **Previous ASCT** | | | |  | >0.999 |
| **Yes** | 1 | 1 |  | 1.0(0.051-1.0) |  |
| **No** | 27 | 24 |  | 0.889(0.719-0.962) |  |
| **Previous CART** |  |  |  |  | >0.999 |
| **Yes** | 3 | 3 |  | 1.0(0.438-1.0) |  |
| **No** | 25 | 22 |  | 0.88 (0.700-0.958) |  |
| **Bridging treatment** |  |  |  |  | >0.999 |
| **Yes** | 3 | 3 |  | 1.0(0.438-1.0) |  |
| **No** | 25 | 22 |  | 0.88 (0.700-0.958) |  |
| **CRS** |  |  |  |  | >0.999 |
| **0-2** | 25 | 22 |  | 0.88(0.700-0.958) |  |
| **≥3** | 3 | 3 |  | 1.0(0.438-1.0) |  |
| **Germline *TP53* mut** |  |  |  |  | >0.999 |
| **Without** | 26 | 23 |  | 0.885(0.710-0.960) |  |
| **With** | 2 | 2 |  | 1.0(0.178-1.0) |  |
| **Del(17p)** (n=20) | | | | | >0.999 |
| **Without** | 5 | 4 |  | 0.8(0.376-0.990) |  |
| **With** | 15 | 13 |  | 0.867(0.621-0.976) |  |
| ***TP53*mut Functional classification Ⅰ** (n=19) | | | | | >0.999 |
| **Non-missense** | 5 | 5 |  | 1.0(0.566-1.0) |  |
| **Missense** | 14 | 12 |  | 0.857(0.601-0.975) |  |
| ***TP53*mut Functional classification ⅠI** (n=19) | | | |  | >0.999 |
| **Non-disruptive** | 12 | 11 |  | 0.947(0.646-0.996) |  |
| **Disruptive** | 7 | 6 |  | 0.857(0.487-0.993) |  |
| ***TP53*mut Functional classification ⅠII** (n=14) | | | |  | >0.999 |
| **Low EAp53** | 7 | 6 |  | 0.857(0.487-0.993) |  |
| **High EAp53** | 7 | 6 |  | 0.857(0.487-0.993) |  |

**Table S4. Summary of adverse events^*^**

|  | **Trial A (N=66)** | | | | **Trial B (N=57)** | | | | ***P* value** |
| --- | --- | --- | --- | --- | --- | --- | --- | --- | --- |
|  | **Any Grade** | **Grade 1-2** | **Grade 3-4** | **Grade 5** | **Any Grade** | **Grade 1-2** | **Grade 3-4** | **Grade 5** |  |
| **CRS** | 60(90.91%) | 36 (54.54%) | 24(36.36%) | 0(0.00%) | 54(94.73%) | 49(85.96%) | 5(8.77%) | 0(0.00%) | 0.0006* |
| **ICANS** | 6(9.09%) | 6(9.09%) | 0(0.00%) | 0(0.00%) | 11(19.30%) | 10(17.54%) | 1(1.75%) | 0(0.00%) | 0.201 |
| **Severe Adverse events** | 30(45.45%) | 25 (37.88%) | 4(6.06%) | 1(1.51%) | 29(50.88%) | 23(40.35%) | 6(10.53%) | 0(0.00%) | 0.607 |
| Secondary Infection | 27(40.91%) | 23(34.85%) | 3(4.54%) | 1(1.51%) | 26(45.61%) | 21(36.84%) | 5(8.77%) | 0(0.00%) | 0.603 |
| Acute heart failure | 4(6.06%) | 4(6.06%) | 0(0.00%) | 0(0.00%) | 4(7.02%) | 3(5.26%) | 1(1.75%) | 0(0.00%) | 0.553 |
| Shock | 6(9.09%) | 5(7.58%) | 1(1.51%) | 0(0.00%) | 4(7.02%) | 3(5.26%) | 1(1.75%) | 0(0.00%) | 0.871 |
| Sepsis | 3(4.54%) | 0(0.00%) | 3(4.54%) | 0(0.00%) | 4(7.02%) | 1(1.75%) | 3(5.26%) | 0(0.00%) | 0.546 |
| **Hematological adverse events** | 66(100%) | 0(0.00%) | 66(100%) | 0(0.00%) | 57 (100%) | 0(0.00%) | 57 (100%) | 0(0.00%) | >0.999 |
| Neutropenia | 63(95.45%) | 0(0.00%) | 63(95.45%) | 0(0.00%) | 57 (100%) | 0(0.00%) | 57 (100%) | 0(0.00%) | 0.103 |
| Lymphopenia | 63(95.45%) | 0(0.00%) | 63(95.45%) | 0(0.00%) | 57 (100%) | 0(0.00%) | 57 (100%) | 0(0.00%) | 0.103 |
| Anemia | 63(95.45%) | 34(51.52%) | 29(43.94%) | 0(0.00%) | 57 (100%) | 5(8.77%) | 52(91.23%) | 0(0.00%) | <0.0001* |
| Thrombocytopenia | 66(100%) | 7(10.61%) | 59(89.39%) | 0(0.00%) | 57 (100%) | 0(0.00%) | 57 (100%) | 0(0.00%) | 0.011* |
| **Chemical laboratory abnormalities** | 54(81.82%) | 45(68.18%) | 9(13.64%) | 0(0.00%) | 52(91.23%) | 48(84.21%) | 4(7.02%) | 0(0.00%) | 0.118 |
| ALT increase | 12(18.18%) | 12(18.18%) | 0(0.00%) | 0(0.00%) | 16(28.07%) | 15(26.32%) | 1(1.75%) | 0(0.00%) | 0.291 |
| AST increase | 9(13.64%) | 9(13.64%) | 0(0.00%) | 0(0.00%) | 17(29.82%) | 16(28.07%) | 1(1.75%) | 0(0.00%) | 0.070 |
| AKP increase | 14(21.21%) | 14(21.21%) | 0(0.00%) | 0(0.00%) | 16(28.07%) | 16(28.07%) | 0(0.00%) | 0(0.00%) | 0.883 |
| γ-GT increase | 10(15.15%) | 10(15.15%) | 0(0.00%) | 0(0.00%) | 14(24.56%) | 14(24.56%) | 0(0.00%) | 0(0.00%) | 0.189 |
| Hypoalbuminemia | 45 (68.18%) | 37(56.06%) | 8(12.12%) | 0(0.00%) | 34(59.65%) | 32(56.14%) | 2(3.51%) | 0(0.00%) | 0.182 |
| Hypertriglyceridemia | 4(6.06%) | 4(6.06%) | 0(0.00%) | 0(0.00%) | 0(0.00%) | 0(0.00%) | 0(0.00%) | 0(0.00%) | 0.059 |
| Hypokalemia | 28(42.42%) | 25(37.88%) | 3(4.54%) | 0(0.00%) | 22(38.60%) | 22(38.60%) | 0(0.00%) | 0(0.00%) | 0.263 |
| Hypochloremia | 35(53.03%) | 35(53.03%) | 0(0.00%) | 0(0.00%) | 21(36.84%) | 20(35.09%) | 1(1.75%) | 0(0.00%) | 0.089 |
| **Other adverse events** | 19(28.79%) | 19(28.79%) | 0(0.00%) | 0(0.00%) | 18(31.58%) | 17(29.82%) | 1(1.75%) | 0(0.00%) | 0.548 |
| Headache | 4(6.06%) | 4(6.06%) | 0(0.00%) | 0(0.00%) | 6(10.53%) | 6(10.53%) | 0(0.00%) | 0(0.00%) | 0.366 |
| Dizziness | 3(4.54%) | 3(4.54%) | 0(0.00%) | 0(0.00%) | 7(12.28%) | 6(10.53%) | 1(1.75%) | 0(0.00%) | 0.240 |
| Tremor | 5(7.58%) | 5(7.58%) | 0(0.00%) | 0(0.00%) | 1(1.75%) | 1(1.75%) | 0(0.00%) | 0(0.00%) | 0.135 |
| Rash | 2(3.03%) | 2(3.03%) | 0(0.00%) | 0(0.00%) | 6(10.53%) | 6(10.53%) | 0(0.00%) | 0(0.00%) | 0.093 |
| Nausea | 12(18.18%) | 12(18.18%) | 0(0.00%) | 0(0.00%) | 7(12.28%) | 7(12.28%) | 0(0.00%) | 0(0.00%) | 0.366 |
| Decreased appetite | 5(7.58%) | 5(7.58%) | 0(0.00%) | 0(0.00%) | 8(14.04%) | 8(14.04%) | 0(0.00%) | 0(0.00%) | 0.245 |

CRS, cytokine release syndrome; ICANS, immune effector cell-associated neurotoxicity syndrome;

*Adverse events that occurred in more than 1 patient after randomization through day 28 are shown. Some patients had more than one adverse event.

**Table S5. Design of sequencing panels covering the coding sequences in *TP53* gene**

| **Sequencing target** | **Target (customized)** | **Target (customized)** | **Target (customized)** | **Target (customized)** | **Target (customized)** | **Target (customized)** | **Whole exome**  **(pre-designed)** |
| --- | --- | --- | --- | --- | --- | --- | --- |
| **Interested region^*^** | Coding region and splice-site | Coding region and splice-site | Coding region and splice-site | Coding region and splice-site | Hotspot region | Hotspot region | Exon and splice-site |
| **No. of targeted genes** | 20 | 28 | 40 | 157 | 108 | 173 | - |
| **Size of customized target** | 87kb | 107kb | 113kb | 230kb | 9kb | 24kb | - |
| **Type of applicable sample** | Fresh/Frozen | Fresh/Frozen or FFPE | Fresh/Frozen | Fresh/Frozen or FFPE | Circulation tumor DNA | Circulation tumor DNA | Fresh/Frozen or FFPE |
| **Sequencing library kit** | AmpliSeqTM Library PLUS for Illumina | | | | | | Agilent SureSelect Human All Exon V6 |
| **DNA input per sample** | 20ng | 20ng | 20ng | 20ng | 30ng | 30ng | 500ng |
| **Expected mean depth** | 1000× | 1000× | 1000× | 1000× | 25000× | 25000× | 200× |
| **No. of detected cases** | 1 | 8 | 6 | 20 | 6 | 3 | 1 |

^*^Covering full coding region (CDS) and splice-site in *TP53* gene.

**Supplemental references**

1. Wang N, et al. Efficacy and safety of CAR19/22 T-cell cocktail therapy in patients with refractory/relapsed B-cell malignancies. Blood. 2020;135(1):17-27.

2. Wei J, et al. Long-term outcomes of relapsed/refractory double-hit lymphoma (r/r DHL) treated with CD19/22 CAR T-cell cocktail therapy. Clin Transl Med. 2020;10(5):e176.

3. Mueller KT, et al. Clinical Pharmacology of Tisagenlecleucel in B-cell Acute Lymphoblastic Leukemia. Clin Cancer Res. 2018;24(24):6175-6184.
